# Recent Advances in Therapy for the Neurodegenerative Disorder Ataxia-Telangiectasia

**DOI:** 10.3390/ijms27167347

**Published:** 2026-08-17

**Authors:** Sam Nayler, Simon Foster, Martin Lavin, David Coman

**Affiliations:** 1 QIMR Berghofer Research Institute, Brisbane, QLD 4006, Australia; 2 Department of Pharmacology, Monash Biomedicine Discovery Institute, Monash University, Clayton, VIC 3800, Australia; 3 School of Biomedical Sciences, University of Queensland, St Lucia, QLD 4072, Australia; 4 Frazer Institute, University of Queensland, Royal Brisbane and Women’s Hospital, Herston, QLD 4029, Australia; 5 Department of Paediatrics, Wesley Medical Research, Auchenflower, Brisbane, QLD 4066, Australia; 6 Department of Metabolic Medicine, Queensland Children’s Hospital, South Brisbane, QLD 4101, Australia; 7 Child Health Research Centre, Faculty of Medicine, University of Queensland, South Brisbane, QLD 4101, Australia; 8 School of Medicine, Griffith University, Gold Coast, QLD 4222, Australia

**Keywords:** ataxia, telangiectasia, ATM, cerebellum, ROS, mitochondria, therapy, A-T, ASO, DNA repair

## Abstract

At present, there is no cure for the human genetic disorder ataxia-telangiectasia (A-T), which is managed by supportive care. This disorder arises due to mutations in the *ATM* (ataxia-telangiectasia mutated) gene and is characterised by a defect in the response to DNA damage, oxidative stress, mitochondrial dysfunction and immune deficiency. The ATM protein is activated by DNA damage, reactive oxygen species (ROS), and a variety of other stimuli, which leads to the phosphorylation or altered cellular localisation of multiple protein substrates that participate in cellular defence pathways. ATM plays a central role in orchestrating cellular defence against stress, which forms a focal point for approaches to treating the symptoms in this disorder. These strategies involve boosting mitochondrial function and dampening the inflammatory response. A more direct approach to treatment is gene therapy, yet the leading method using Adeno-Associated Virus (AAV) is hampered by the large size of the ATM gene itself. The use of antisense oligonucleotides (ASO) as an alternative gene therapeutic approach to treat patients has been increasingly utilised. However, only certain mutations fit the criteria for ASO-based intervention, which encourages rescue through readthrough of premature truncation mutations. For this reason, small-molecule-based therapies addressing the multi-system nature of the disease are urgently required. We address these different approaches to therapy and the outcome of numerous recent clinical trials with A-T patients, as well as ongoing research that has potential to lead to therapy.

## 1. Introduction

**Figure 1:**
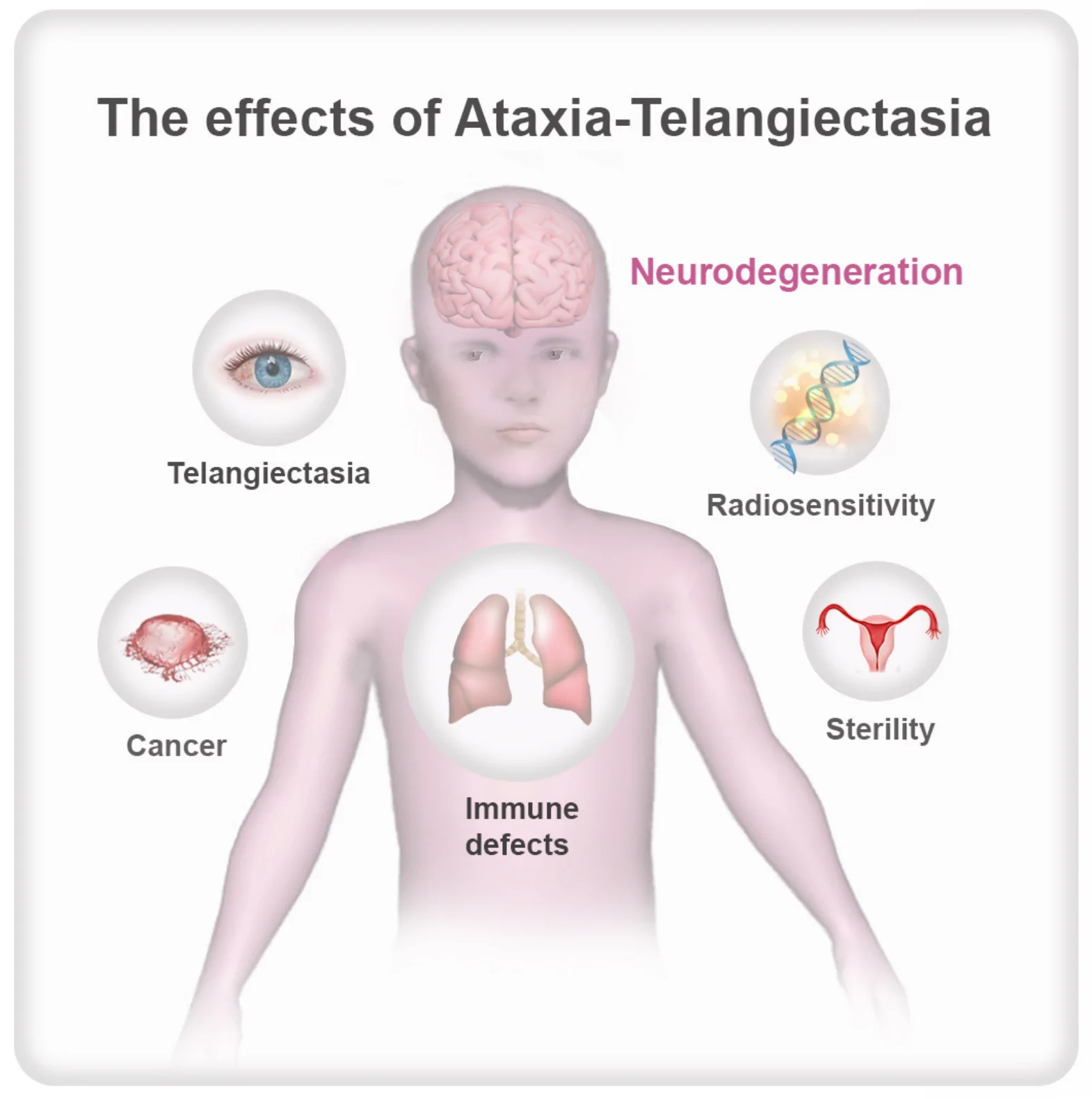
ATM is a multisystem syndrome featuring telangiectasia, predisposition to cancer, radiosensitivity, sterility and profound neurodegeneration.

Ataxia-telangiectasia (A-T, OMIM 208990) is an autosomal recessive genetic disorder caused by mutations in the *ATM* gene (OMIM 607585) [1]. This syndrome is characterised by progressive cerebellar degeneration, oculocutaneous telangiectasia, bronchopulmonary disease, elevated risk of lymphoid and other forms of cancer, immunodeficiency, infertility and clinical radiosensitivity [2,3] (Figure 1). Consequently, the majority of patients succumb to either complications associated with lung disease or cancer [4]. A-T also features insulin-resistant diabetes and chromosomal instability, cell cycle defects and increased sensitivity to ionising radiation of cells in culture. The establishment of a central role for ATM in the DNA damage response provided an explanation for the hypersensitivity to radiation and chromosomal instability observed in A-T cells and could also contribute to characteristics such as neurodegeneration, immune defects, meiotic recombination defects and increased cancer risk seen in patients [5].

**Figure 2:**
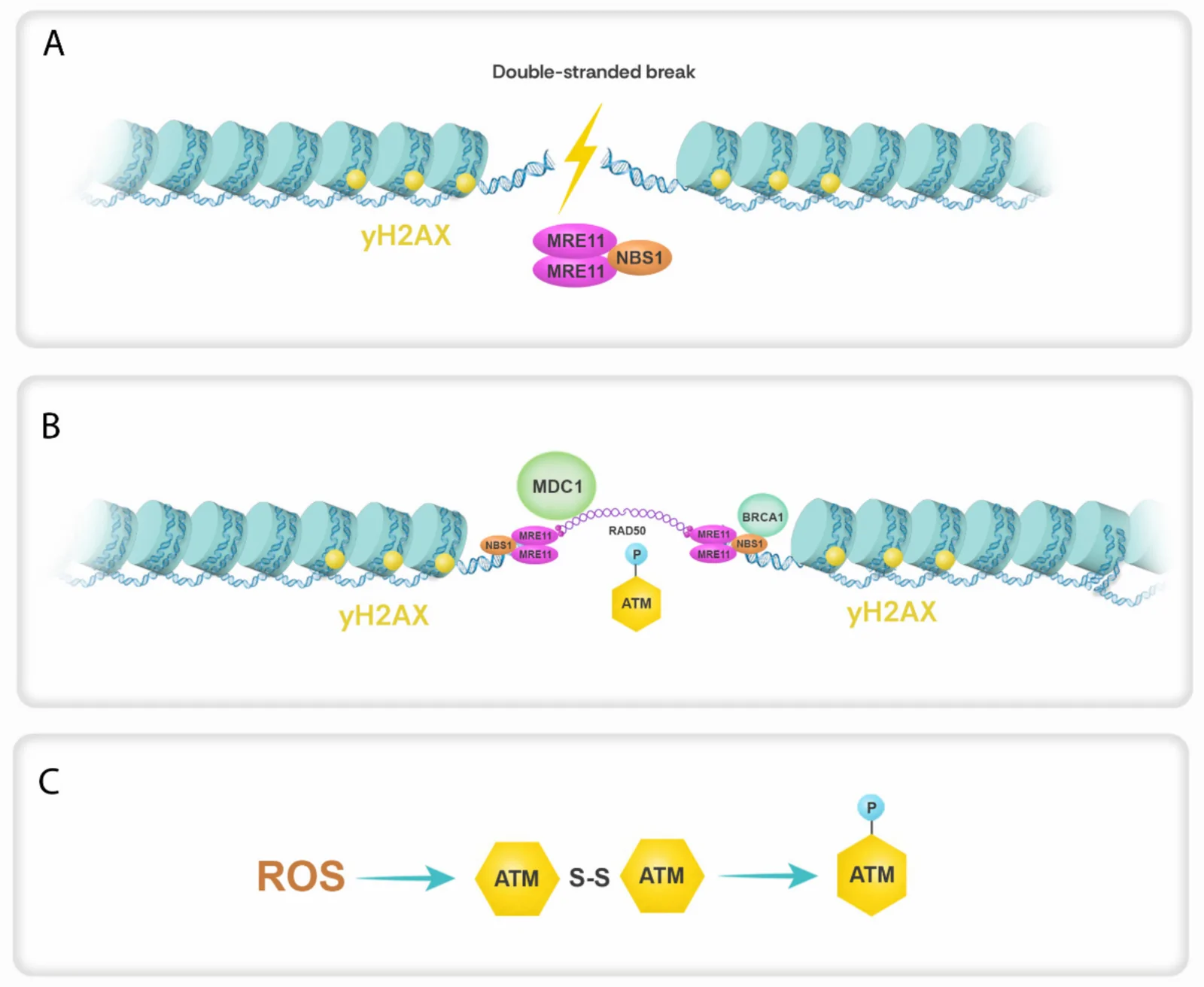
Activation of ATM. (**A**) Double-strand breaks recruit and activate MRE11/NBS1 prior to ATM activation. (**B**) ATM autophosphorylation after recruitment by the MRE11-RAD50-NBN complex to sites of DNA double-strand breaks. (**C**) ATM is also activated by a separate mechanism by ROS.

ATM is activated by autophosphorylation at several sites and subsequent monomerisation of an inactive dimeric form, after recruitment by the MRE11-Rad50-NBN complex to sites of DNA double-strand breaks [6,7] (Figure 2). ATM then phosphorylates members of the MRE11 complex and, in turn, a multitude of proteins that control major cellular processes including DNA repair, cell cycle regulation and transcription. The role of ATM in the DNA damage response and its relationship to neuronal degeneration has been reviewed extensively elsewhere [4,8]. ATM is also activated by a separate mechanism by reactive oxygen species (ROS) [9]. ROS are generated by a number of different processes in the cell but primarily by mitochondria [10]. This activation is independent of the MRE11 complex and involves a redox-formed disulfide linkage between cysteine residues on each of two ATM monomers displacing an autoinhibitory loop [11]. Oxidative stress is also important in the activation of ATM when glycolysis is inhibited and also due to other sources of cellular damage, including mechanical stress [12,13].

The major neuropathology observed in A-T is degeneration or atrophy of the cerebellar vermis and hemispheres, involving both Purkinje cells and granule neurons, and this progresses with age [7]. Greater than 25% of patients with A-T develop chronic lung disease (and if not managed can lead to bronchiectasis, recurrent pneumonia, lung fibrosis and interstitial lung disease [14]). Immune deficiency is common in A-T, including reduced levels of immunoglobulins, lymphopenia, reduced antigen receptor repertoire, and cutaneous granulomas [15,16]. Control of eye movement is impaired and includes oculomotor apraxia, nystagmus and abnormal saccades [17]. Dysphagia gradually develops in patients due to neurological decline, leading to difficulty in feeding and swallowing [18]. Non-alcoholic fatty liver disease is a recently recognised part of the disease natural history [19,20]. Abnormalities in growth, sleep initiation and maintenance, cognition as well as sensitivity to radiotherapy are also reported in A-T patients [21,22].

Here we explore recent developments in A-T clinical trials and also supporting findings from laboratory-based disease models. As A-T is a multisystem disease, different therapeutic modalities may preferentially target particular organ systems, which can have implications for treatment. Understanding the complex interactions of drugs in specific organs, such as the liver and lungs, as well as the periphery/neuroimmune axis, will be crucial to treating the symptoms of A-T and minimising off-target effects. Finally, we acknowledge emerging clinical methodology for treating other central nervous system (CNS) disorders with clinical overlap to A-T.

## 2. Therapeutic Targeting of the CNS

**Figure 3:**
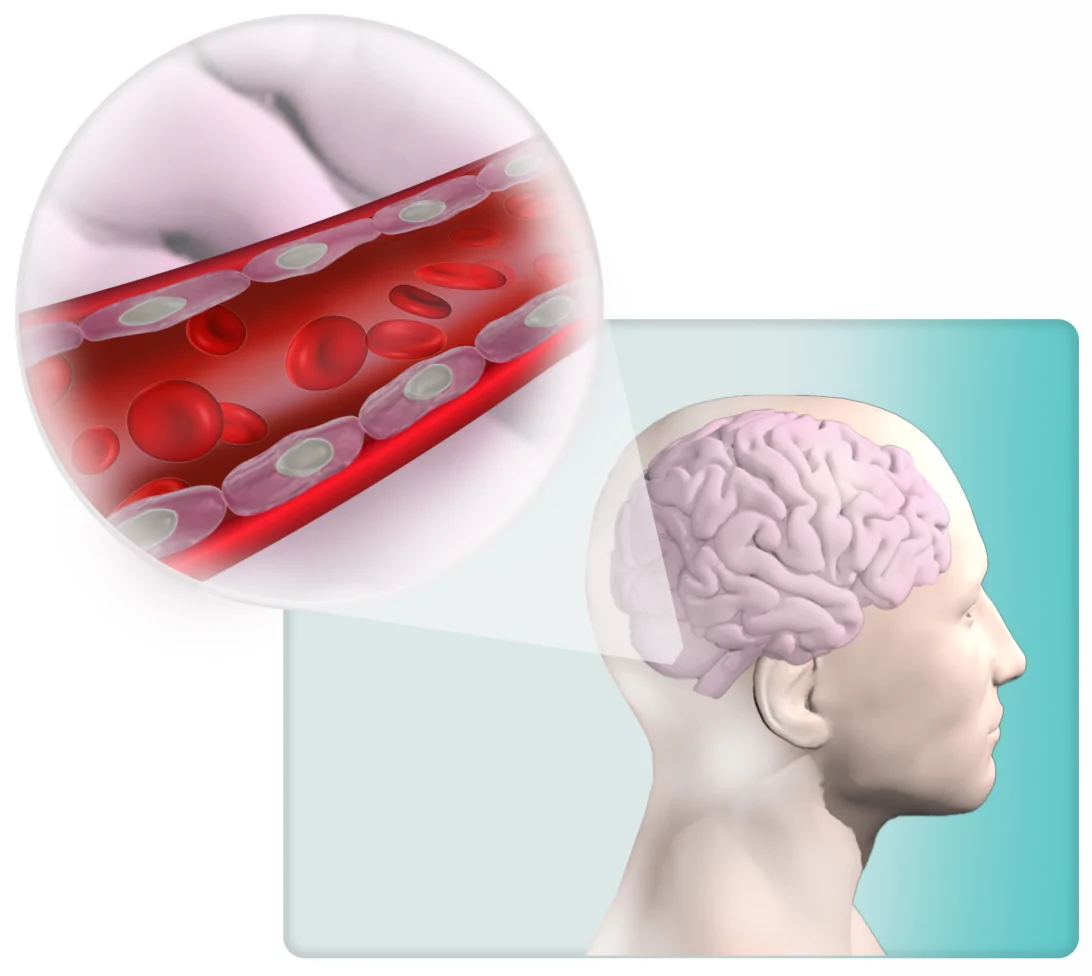
CNS targeting drugs must be able to cross the blood–brain barrier.

Laboratory research has revealed context-specific roles for ATM in the CNS, particularly concerning neurotransmission, neuroinflammation, and metabolic regulation/energy homeostasis. The multiple functions of ATM may offer opportunities to identify candidates for targeting in therapeutic interventions. However, despite decades of work, the precise mechanisms underlying pathology in A-T remain elusive. This has been hampered by animal models that do not completely recapitulate the neurological phenotype, although recent progress with an ATM-deficient macaque model shows compelling evidence of key overlapping phenotypes with the human manifestation (growth retardation, immune deficiency, telangiectasia and motor dysfunction) [23]. Targeting cells within the CNS requires therapeutic agents capable of crossing the blood–brain barrier (BBB) (Figure 3). ATM kinase inhibitors, such as AZD1390, have been specifically developed as brain-penetrating agents, demonstrating the technical feasibility of CNS targeting, albeit in this case to treat cancer. Repurposing of drugs with known brain-penetrance is also another option [24].

**Figure 4:**
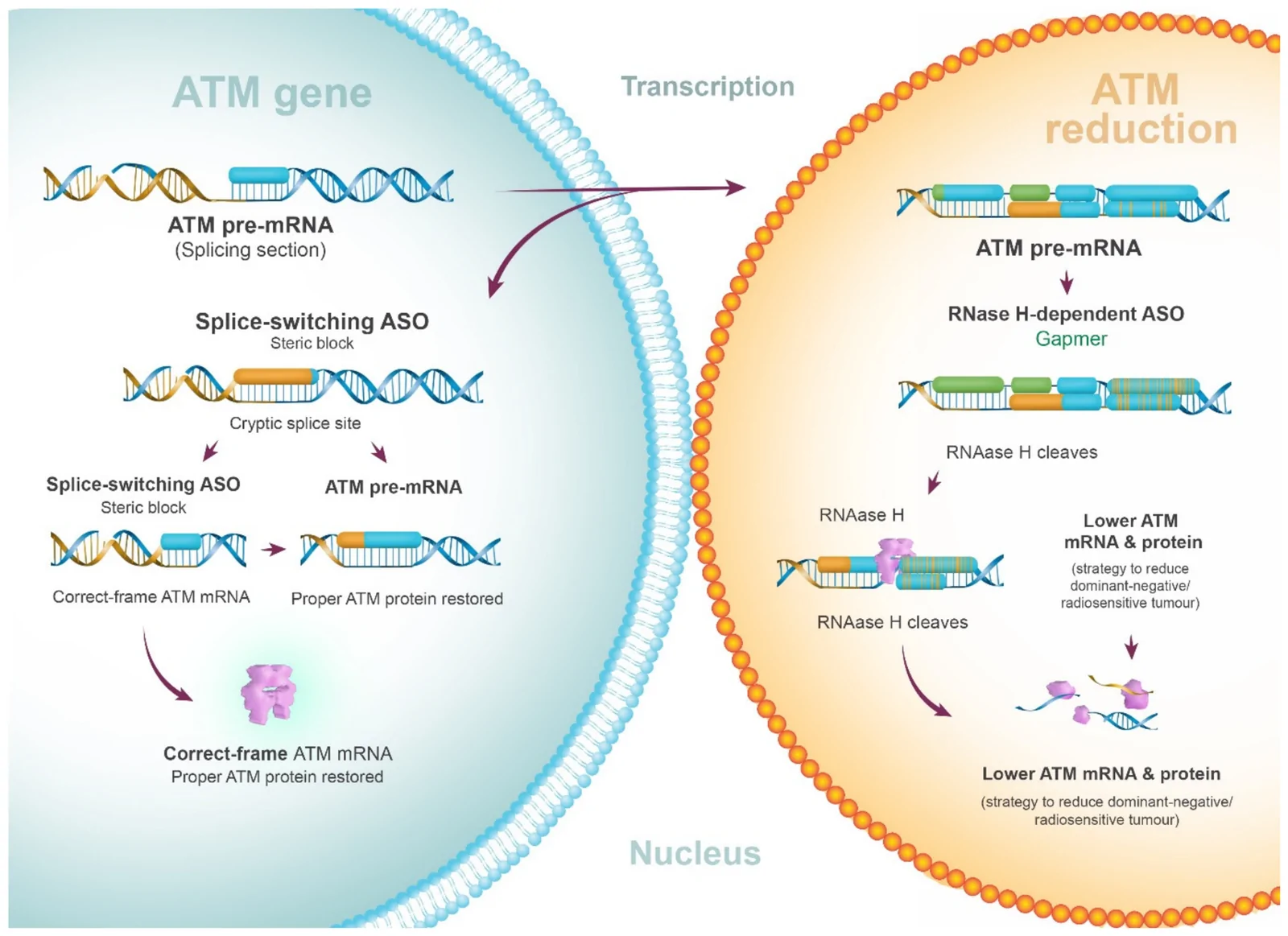
Schematic depicting ASO-mediated therapies for A-T. Splice-switching ASOs are designed to correct out-of-frame ATM mutations and restore functional protein expression (left-hand side). RNAse H-dependent ASOs have also been utilised to hybridise to ATM mRNA and ablate protein as strategies for ATM knock-down experiments or for tumour sensitisation driven by dominant-negative ATM activity (right-hand side).

Antisense oligonucleotide (ASO) therapy is inherently well-suited for CNS delivery, as intrathecal injection allows ASOs to readily distribute within the CNS, including the cerebellum (Figure 4). In the last ten years, there has been significant headway with respect to clinical translation of therapeutic ASOs. Eight ASOs have been granted marketing approval by the United States Food and Drug Administration (US FDA), following the pioneering ASO drug fomivirsen (Vitravene, approved in 1998) [25]. ASOs targeting splice site modulation have been developed for therapy of inborn errors of metabolism, because of their ability to redirect aberrant splicing mutations, thus recovering normal expression of transcripts, and correcting the deficiency of functional proteins. ASOs with splice-switching capacity show promise in restoring functional ATM protein expression. Researchers recently developed a groundbreaking framework to systematically identify splice-switching treatments for A-T [25,26]. Preclinical development identified ASOs targeting specific pathogenic variants, such as c.5763-1050A>G (a UK founder variant) and c.7865C>T, with up to 9% of individuals identified as possibly amenable to ASO splice modulation [26]. ASO treatment successfully restored ATM signalling in patient fibroblasts, evidenced by the rescue of radiation-induced phosphorylation of key downstream effectors like pP53 and pKAP1. One ASO, AT008 (atipeksen), showed preclinical efficacy and was introduced into an experimental N-of-1 trial [27]. Ongoing questions about the required dosage for ATM restoration, delivery, timing and long-term safety are yet to be explored. While correction of ATM expression via ASO is promising, it is not a universally applicable method, with patient/mutation-specific ASOs being required (Figure 4 shows two different modalities for ASO use related to modulation of ATM activity).

## 3. Therapeutics Targeting Mitochondria

The understanding of A-T has expanded significantly beyond DNA damage repair to include intricate roles for ATM in metabolism, mitochondrial health, and inflammation, leading to new therapeutic approaches targeting these overlapping systems termed ‘immunometabolism’. Cellular energy production systems must be dynamic and flexible, given changing requirements for energy demand across the course of life and in different cell and tissue types. This may be driven by external stimuli or innate energy requirements (i.e., neuronal cells expend tremendous amounts of energy regulating synaptic operation, membrane potential and ion channel homeostasis) and immune cells must be able to rapidly sustain energy production in response to an immune challenge. A neuron at rest houses one billion molecules of ATP—firing of an action potential is estimated to require hydrolysis of between 10–100 million ATP molecules to re-equilibrate membrane potential [28]. This is critical in the case of A-T, since cells lacking ATM replenish ATP poorly following surges in energy demand [29]. A major mechanistic finding links ATM loss to profound mitochondrial dysfunction and impaired energy response, particularly critical in high-energy demand neurons like Purkinje cells. Laboratory findings show ATM acts as a sensor of ATP insufficiency; when ATP is depleted, ROS are generated, which activates ATM, independent of DNA damage. Activated ATM then phosphorylates Nuclear Respiratory Factor 1 (NRF1), facilitating NRF1 dimerisation, nuclear translocation, and subsequent up-regulation of genes necessary for the electron transport chain and mitochondrial activity. In ATM-deficient cells, this crucial response fails, resulting in diminished ATP reserve capacity. Crucially, ectopic expression of a phosphomimetic mutant of NRF1 (T259E) rescued bioenergetic defects in Atm−/− neurons, validating NRF1 as a key downstream effector [29].

In addition, microglia (the resident brain macrophage) are metabolically flexible and will markedly change their metabolic activity in response to inflammation [30]. Microglia predominantly rely on oxidative metabolism at rest and switch to glycolysis following inflammatory activation. ATM is involved in controlling responses to oxidative stress and regulating glucose metabolism as part of this, and has generally been described in many settings; however, defining critically impaired and targeted energy metabolism deficiencies in the cerebellum and other organ systems compromised in A-T requires further work [30,31].

**Figure 5:**
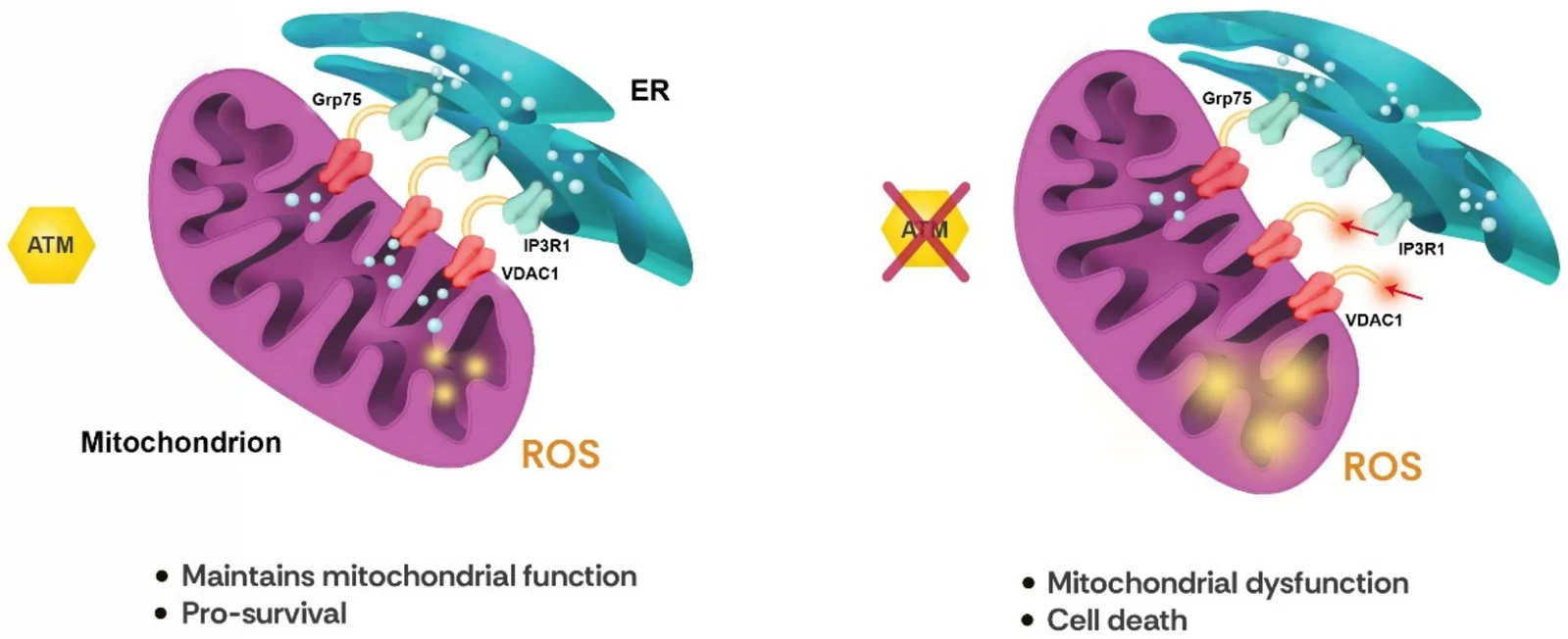
ATM regulates ER–mitochondrial stress signalling and mitochondrial homeostasis. Schematic illustrating the role of ATM in maintaining mitochondrial function during cellular stress (**left**), and impaired IP3R1-VDAC1-mediated calcium regulation in the absence of ATM (**right**).

Under normal conditions (Figure 5, left), ATM supports communication between the endoplasmic reticulum (ER) and mitochondria at contact sites involving GRP75, IP3R1, and VDAC1, facilitating controlled calcium transfer and preserving mitochondrial metabolism while limiting excessive reactive oxygen species (ROS). In the absence of ATM (Figure 5, right), dysregulated ER–mitochondrial signalling disrupts calcium handling, leading to mitochondrial dysfunction, increased ROS accumulation, and activation of cell death pathways. This model highlights a key role for ATM in coordinating stress responses that preserve mitochondrial integrity and cellular survival.

Increased rates of mitochondrial respiration in Atm−/− cerebella represent early evidence that the cerebella of these mice function under energetic stress [32]. Subsequent studies revealed a link between the oxidative stress phenotype in A-T cells and intrinsic mitochondrial dysfunction [33]. In the same year, Eaton et al. provided evidence that disruption of ribonucleotide reductase and mitochondrial homeostasis contributed to the complex pathology of A-T [34]. ATM loss can lead to intrinsic mitochondrial abnormalities in thymocytes, elevated levels of ROS, increased aberrant mitochondria, high cellular respiratory capacity and decreased mitophagy [34]. Furthermore, decreasing mitochondrial ROS with the mitochondrial-targeted antioxidant MitoQ in Atm(+/−)/ApoE(−/−) mice prevented abnormalities associated with metabolic syndrome [35]. Likewise, overexpression of catalase targeted to mitochondria as a means of reducing ROS alleviated A-T-related pathology in Atm−/− mice [36]. Reduced mitochondrial DNA integrity is associated with defective repair of mitochondrial DNA, reduced levels of Ligase 3 protein, and is implicated in mitochondrial dysfunction in Atm−/− mice [37]. These studies represent only some of the reports on mitochondrial dysfunction in A-T cells. As is obvious from this body of literature, elevated ROS is a consistent characteristic of A-T cells. It is also evident that ROS activates ATM by a mechanism distinct from that after DNA damage [6,9]. The localisation of ATM to mitochondria [12,38] and its activation by oxidative stress in mitochondria further emphasises the importance of mitochondria to the A-T phenotype [39].

### 3.1. NAD+ and Precursor Intervention

**Figure 6:**
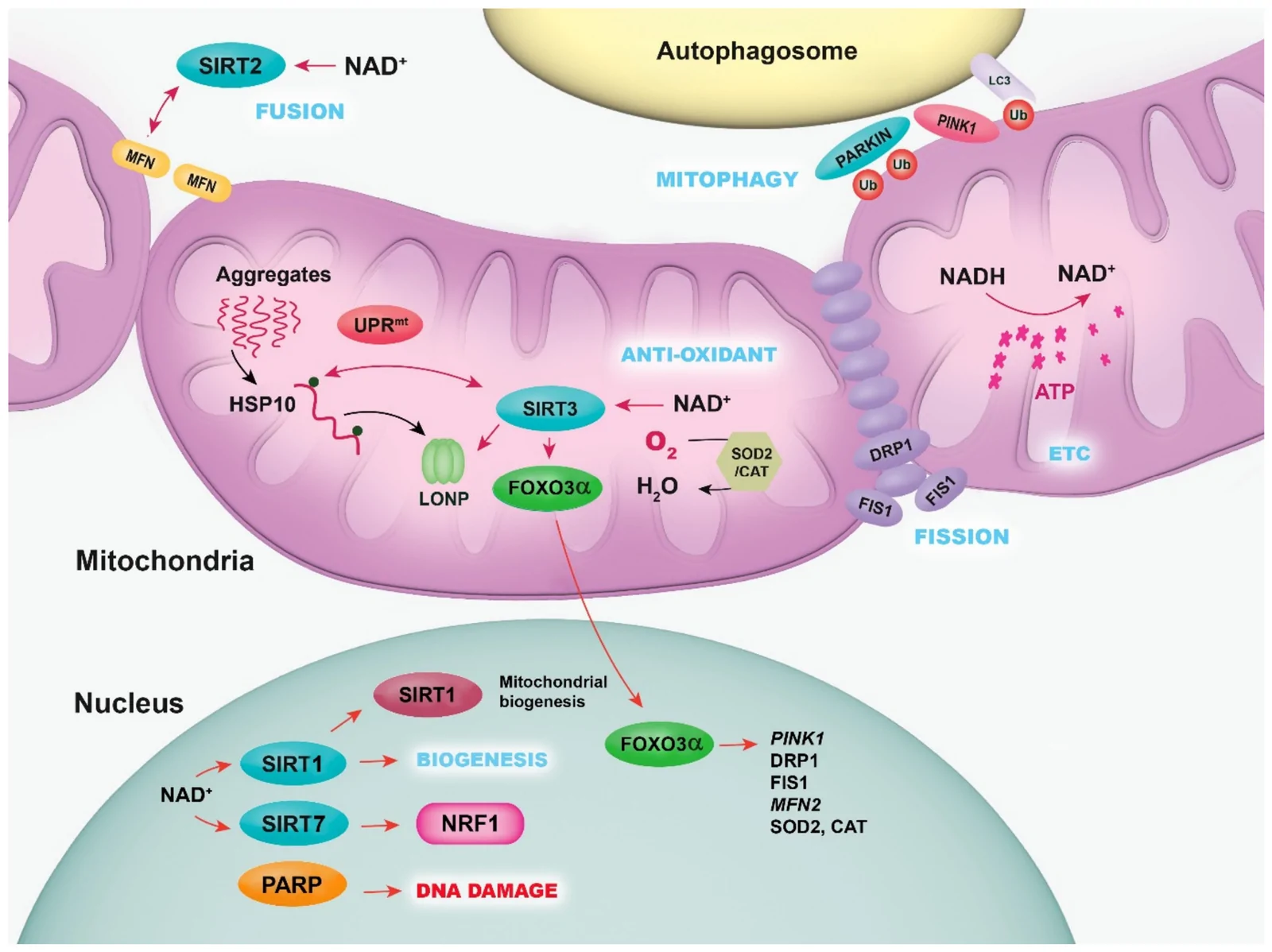
ATM-NAD^+^ signalling regulates mitochondrial quality control and stress responses. Schematic illustrating mitochondrial and nuclear pathways influenced by ATM-dependent stress signalling.

A variety of antioxidants have been demonstrated to correct different aspects of the A-T phenotype [40,41,42], but whether that can be extrapolated to therapy for patients remained unresolved. One candidate stood out, being NAD+ (nicotinamide adenine dinucleotide). A study employing primary rat neurons, C. elegans and Atm−/− mice [43] showed that loss of ATM caused mitochondrial dysfunction and compromised mitophagy due to NAD+ insufficiency. Repletion with NAD+ or its precursors reduced the severity of A-T neuropathology, normalised neuromuscular function, delayed memory loss and extended lifespan in both animal models. NAD+ mitigated both the DNA repair defect and mitochondrial dysfunction. The authors concluded that persistent DNA damage activates signalling from the nucleus to the mitochondrion, a pathway which may play an important role in normal aging. They subsequently showed that supplementation with the NAD+ precursor nicotinamide riboside (NR) improved lymphoid lineage potential in aged mice [44,45,46]. NR treatment was shown to prevent senescence in fibroblasts and improve motor function in Atm−/− mice, suppressing neurodegeneration and neuroinflammation [44]. Within mitochondria, NAD+-dependent sirtuins, particularly SIRT3, promote antioxidant defences through FOXO3α and activation of SOD2 and CAT, limiting ROS and associated damage. Mitochondrial integrity is maintained through protein quality control (UPRmt, HSP10, LONP), mitochondrial dynamics (MFN, DRP1, FIS1), and PINK1-PARKIN-mediated mitophagy. In the nucleus, SIRT1/SIRT7-NRF1 signalling regulates mitochondrial biogenesis, while PARP activation during ATM-driven DNA damage responses consumes NAD^+^. However, it is evident that NAD+ affects a number of critical pathways (depicted in Figure 6) other than mitochondrial homeostasis, including RNA processing, metabolic switching, regulated by sirtuins, nucleotide and amino acid synthesis and DNA replication, all of which broaden the role of this cofactor in influencing biological processes [47].

The success of NR in in vitro cell culture and animal models suggested that it would be a potential candidate for treatment of patients with A-T. Veenhuis et al. [48] described an unblinded open-label, proof-of-concept study with 24 patients treated with NR (25 mg/kg body weight each day) for four months followed by a two-month period without the drug. Metabolomic analysis confirmed increased plasma levels of NR metabolites during the treatment period. Improvement in ataxia scores measured by both the Scale for the Assessment and Rating of Ataxia (SARA) and the International Cooperative Ataxia Rating Scale (ICARS), decreasing to 2.4 and 10.1, respectively [48]. These improvements disappeared after NR withdrawal. In laboratory measurements, serum IgG increased in patients with classic A-T while immunoglobulin concentrations were normal at baseline in variant A-T patients. Limitations of the study included lack of randomisation and placebo control, as well as small numbers accounting for inability to observe dose effects.

An additional study was established to investigate the safety and benefits of long-term supplementation with NR in patients [49]. Among 13 patients enrolled for the study, 10 patients completed 18 months of NR treatment. NR was well tolerated in patients, and total scores in the neuromotor test panels improved for all but one patient, primarily in co-ordination subscores and eye movement. No clinically significant changes in a set of 10 liver or kidney biomarkers were seen, nor were levels of total immunoglobulins or subclasses thereof. Neurofilament light (NfL) chain levels were elevated in patients as previously reported [48,50]. Use of NR with a single young patient (3.5 years old) with recurrent infection replicated these findings, showing that SARA scores decreased from 27 to 9 points and mean total score for Gross-Motor-Function increased from 61 to 78% [51]. Overall, NR supplementation represents an easy, safe and non-invasive intervention that provides evidence for neuromotor improvement in patients with A-T. Its efficacy as a therapeutic agent requires large multicentre, international placebo-controlled clinical trials.

### 3.2. Triheptanoin (Dojolvi^®^)

**Figure 7:**
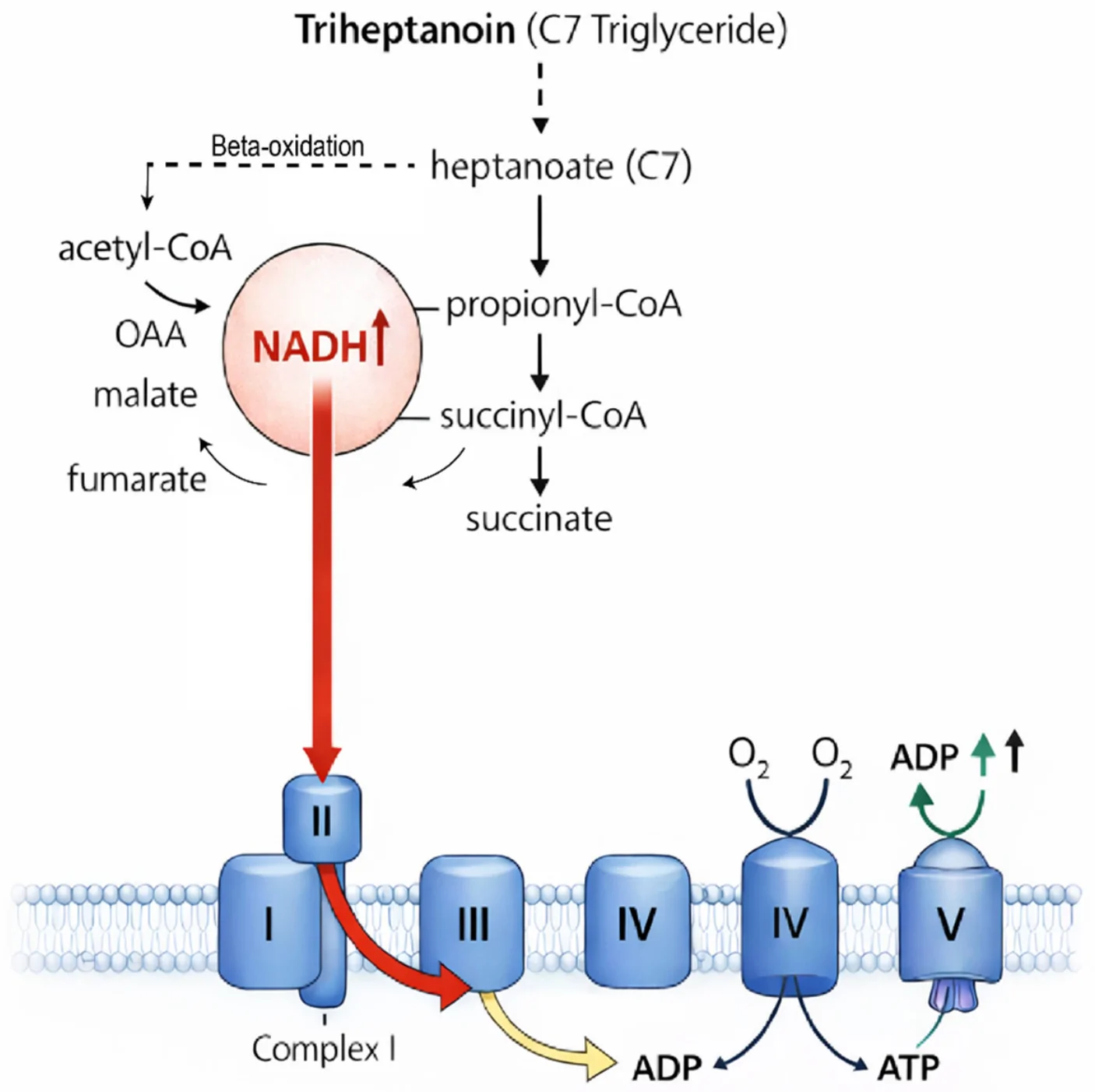
Triheptanoin supplies anaplerotic substrates to support TCA cycle metabolism. Schematic illustrating the metabolic fate of triheptanoin, a triglyceride composed of C7 fatty acids. Following hydrolysis, heptanoate undergoes mitochondrial β-oxidation to generate acetyl-CoA and propionyl-CoA. Propionyl-CoA is subsequently converted to methylmalonyl-CoA and then succinyl-CoA, replenishing TCA cycle intermediates through anaplerosis. These reactions support production of key metabolites including succinyl-CoA, malate, oxaloacetate, and downstream glutamate, thereby sustaining mitochondrial energy metabolism.

The medium-chain odd-chain triglyceride, triheptanoin, composed of three heptanoate (seven-carbon fatty acids) linked to glycerol, has anaplerotic capability, in that it can supply intermediates for the TCA cycle [52] (Figure 7). This compound has been approved for treatment of both paediatric and adult patients with long-chain fatty acid oxidation disorders (LC-FAOD, Ultragenyx Pharmaceutical Inc, DOJOLVI^®^, https://www.dojolvi.com/ (accessed on 6 August 2026). Efficient energy production is provided by three mitochondrial metabolic pathways: the Krebs tricarboxylic citric acid (TCA) cycle, β-oxidation and oxidative phosphorylation (OXPHOS). In the case of LC-FAOD, a deficiency in TCA cycle intermediates occurs as a result of depletion of odd-chain carbon compounds [53]. A recent report confirms the long-term efficacy of triheptanoin for patients with LC-FAOD [54]. A-T cells may be hypersensitive to metabolic stress because of reduced ER-mitochondrial signalling and Ca^2+^ transfer in response to nutrient deprivation. This can result in mitochondrial dysfunction, indicating that A-T cells might respond positively to treatment with triheptanoin [53]. The triheptanoin metabolite heptanoate showed enhancement of ER-mitochondrial contacts, increased flow of calcium between ER and mitochondrion, enhanced mitochondrial function and mitophagy and increased resistance of ATM-deficient cells and cells from A-T patients to metabolic stress-induced killing [55]. The greater reliance on aerobic glycolysis in A-T cells, as shown by increased lactate dehydrogenase (LDHA), accumulation of lactate, and reduced levels of both acetyl-CoA and ATP, was all restored by heptanoate, supporting the use of the parent compound for patient treatment.

### 3.3. Triheptanoin Clinical Trial

On the basis of these compelling preclinical data, a Phase 2a/b trial of triheptanoin with a three-arm placebo-controlled dose-escalation design was conducted in patients with A-T (NCT04513002). The primary outcome was cell death in respiratory epithelial cells. Key secondary outcomes included SARA, ICARS, speech and swallowing function, and novel biomarker discovery [56]. In total, 31 participants with A-T, aged from 4 to 37 years (median 16-years) were enrolled. For maximum dose vs. placebo or no dose, significant improvements were observed for primary outcome percent nasal epithelial cell death (mean difference (MD) = −9.7%, 95% confidence interval (CI) −16.0, 4.6). The SARA subscale kinetic function improved (MD = −5.8, 95% CI −10.4, −1.2), as did ICARS subscales gait (MD = −0.5, 95% CI −0.9, −0.1) and fine motor disturbance (MD = −2.7, 95% CI −4.3, −1.1). Speech intelligibility (MD = −12.8, 95% CI −21.2, −4.3) and swallowing safety (−0.9, 95% CI −1.6, −0.3) improved. Improvements in mitochondrial function were observed after triheptanoin administration. Biomarkers neurofilament light chain and interferon signature stimulated gene scores improved over the course of the trial and suggest that these will be useful for monitoring of disease progression and treatment response in patients.

### 3.4. N-Acetyl-L- Leucine

N-acetyl-DL-leucine (ADLL) is an acetylated derivative of the amino acid leucine and is marketed under the trade name of Tanganil (Pierre Fabre Laboratories, Castres, France) and has been used for the treatment of vertigo for many years [57]. The therapeutic effects appear to be due to the more pharmacologically active metabolite L-enantiomer (NALL). While it remains uncertain how acetyl-DL-leucine affects the brain it has been shown to restore membrane potential in vestibular neurons, possibly by direct interactions with membrane phospholipids affecting channel activity [58]. NALL has been shown to reduce neuroinflammation in the cerebellum and other brain regions possibly by activating autophagy and removing neurotoxic components [59]. Marked reduction in the expression of the pro-inflammatory cytokine IL-1β was observed in brain injured mice treated with NALL and these animals showed improvement in both motor skills and memory [59,60]. In relation to mechanism, a mouse model of Sandhoff’s disease treated with NALL normalized glucose and glutamate metabolism, increased autophagy and also levels of superoxide dismutase (a scavenger of ROS) [61]. Altered glucose and anti-oxidant metabolism, as well as improvements in mitochondrial function were also observed in a mouse model of Niemann-Pick type C treated with the same compound [61]. Metabolic conversion to L-leucine may contribute to neurotransmitter synthesis and neuronal signalling leading to neuroprotective effects [62].

### 3.5. Clinical Trials Using ADLL

In a small study of six A-T patients treated with ADLL, a significant effect on cerebellar signs and symptoms was found including fine motor function, cerebellum-specific ocular motor deficits decreased in addition to an increase in quality of life in patients [63]. A multinational, multicentre, open-label, rater-blinded prospective Phase II study using ADLL for the treatment of A-T is underway. A case study employing ADLL with a nine-year-old patient with Multiple Sulfatase Deficiency showed that it was well tolerated and improved ataxia symptoms and quality of life [64]. The first placebo-controlled, randomized clinical trial exploring NAL’s potential effects for treating A-T, failed to improve motor function significantly but had reduced patient nausea and constipation [65]. In contrast, in a randomized, placebo-controlled, double-blind crossover phase, an extension of a phase 2B IB1001-303 (NCT03759678) trial, NALL was shown to slow disease progression in patients as measured by SARA scores (NeurologyLive, Monroe Township, NJ, USA, 7 October 2025). While early evidence for the use of NALL appears positive, it is evident that larger randomized trials are necessary to confirm its efficacy in treating patients with neurological disorders in general and A-T specifically.

## 4. Addressing the Inflammatory Phenotype in A-T

A-T patients suffer from immunodeficiency; both T-cell and B cell immunodeficiency causes low IgG levels as well as reduced CD4+CD8+ mature thymocytes [66]. This gives rise to defective immune responses manifested in recurrent sinopulmonary infections, which can be attributed to problematic swallowing (dysphagia) and aspiration; many patients require immunoglobulin replacement therapy. ATM is involved with correct development and function of the immune system [67]. Furthermore, because of the broad nature of ATM’s role in protection, multiple cell systems throughout the body are compromised, resulting in chronic inflammation. Prominent and widespread activation of microglia, the immune sentinel of the brain, was shown recently in cerebella of AT patients [68]. This suggested that cell-intrinsic activation of microglia was upstream of and influencing the death of Purkinje and granule cells due to secretion of neurotoxic factors.

Independent research indicates a chronic inflammatory state in A-T patients, marked by elevated levels of proinflammatory cytokines such as IL-6 and IL-8, with IL-8 elevation correlating with increased malignancy risk and mortality [69,70,71]. Dexamethasone treatment, besides its effect on ATM splicing, is known to promote the NRF2-mediated antioxidant response in A-T cells [72]. Given the pro-inflammatory response that characterises A-T cells and reduced capacity to counter oxidative stress, these cells represent good candidates for treatment with steroids. Dexamethasone treatment of A-T cells has been shown to lead to a non-canonical splicing event giving rise to an alternate transcript and, as a consequence, an ATM variant referred to as miniATM. This shortened ATM protein is capable of partially rescuing the A-T phenotype through kinase ability retention [73]. Genes altered after this treatment include those controlling metabolic processes and multiple pathways relevant to A-T [74], highlighting the signalling role of ATM in inflammation. In this context, the steroid betamethasone has been shown to suppress inflammation and enhance oxidative stress pathways [75].

### A-T Clinical Trials Using Steroids

Early trials with oral betamethasone were shown to reduce ICARS scores, reducing ataxia symptoms by 28% in an intent-to-treat population and by 31% in a pre-protocol population [76]. However, since a significant percentage of patients became steroid-dependent and developed adverse side effects, scheduling and dosing presented impediments to this approach. To overcome the side effects of long-term administration of oral steroids, a method was developed for encapsulation of dexamethasone sodium phosphate into autologous erythrocytes called EryDex [77]. EryDex enables slow release of the steroid in patients, which leads to a significant improvement of the neurological symptoms, minimising side effects. In an extension of this study, four patients experienced neurological improvement, and this method of delivery proved to be safe and well-tolerated [78]. Furthermore, the shorter ATM transcript (ATMdexa1, miniATM) was found in peripheral blood mononuclear cells (PBMCs) in patients treated with EryDex, demonstrating a correlation with clinical response after low-dose dexamethasone [78].

In a more recent development, Quince Therapeutics have developed an eDSP (formerly EryDex) platform using ground-breaking pharmacokinetic modelling to reveal a rapid peak in the drug at short times after infusion, followed by a 20–30 day therapeutic window without accumulation [79]. Maureen Roden (Quince Therapeutics, South San Francisco, CA, USA) recently described an ATTeST Phase 3 trial demonstrating that eDSP was well tolerated and slowed neurological deterioration across all age groups for A-T patients [80]. Following this, a Phase 3 clinical trial “Evaluate the Neurological Effects of EryDex on Subjects with A-T” (NEAT) (NCT06193200) was conducted but failed to meet primary and secondary endpoints, prompting Quince Therapeutics to discontinue any further eDSP research in A-T. In a separate study, treatment with eDSP was not related to metabolic or endocrine problems or adrenal insufficiency and patients’ growth in height was preserved. Low serum iron was noted in some patients, requiring further investigation [80]. An offshoot of these studies was the detection of a miniATM protein after dexamethasone exposure and its potential role as a disease biomarker (Michele Menotta, University of Urbino, Urbino, Italy).

## 5. Ongoing Research with Small Molecule Therapies for A-T

### 5.1. Modulation of ATM and Its Protein Kinase Activity

Restoring ATM function, even partially, is a key therapeutic goal, especially given that ATM protein and residual kinase activity correlate with a milder phenotype and increased lifespan in patients [81]. Progress in restoring ATM kinase activity focuses on leveraging the protein’s central role as a serine-threonine protein kinase in cellular signalling, particularly DNA repair. Mutation in the *ATM* gene leads to radiosensitivity and predisposition to cancer. Previously, full-length *ATM* cDNA has been employed to correct the A-T cellular phenotype, including radiosensitivity, induced chromosomal abnormalities and cell cycle checkpoint defects [82]. For A-T itself, recent laboratory and clinical work has focused on gene-based therapies and drug repurposing aimed at restoring ATM activity. The large size of the *ATM* coding region (9.2 kb) poses a challenge for traditional gene therapy. Because of the nature of the progressive ataxia, it will be necessary to deliver the ATM gene into Purkinje cells or other supportive cells in the cerebellum at a time-critical juncture, identification of which remains a challenge.

CRISPR/Cas9-assisted gene correction has enabled restoration of functional ATM alleles in induced pluripotent stem cells from an A-T patient carrying compound heterozygous exonic missense/frameshift mutations, and from a patient with a homozygous splicing acceptor mutation of an internal coding exon [83]. Correction of one allele restored expression of ~50% of full-length ATM protein and its activation by DNA damage, phosphorylation of downstream substrates, reduced radiosensitivity and mitochondrial ROS production, as well as oxidative-stress-induced apoptosis. This approach will enable further studies on the role of ATM in ageing and the pathogenesis of A-T.

A recently described novel approach is the use of split-intein mediated protein transplicing, discussed by Guiliana Giardino (University of Naples, Napoli, Italy) and Margherita Rossi (IRCCS San Raffaele Scientific Institute, Rome, Italy) [80]. This approach involves expressing two fragments of ATM in independent lentiviral vectors. Dual-intein lentiviral vectors were designed that undergo trans-splicing to enable full-length protein reconstitution in fibroblasts. Full-length and functional ATM could be derived using this approach and shown to restore the ability of ATM-deficient cells to carry out ATM-dependent phosphorylation events. The approach was also successfully used in hematopoietic stem cells in ATM-deficient mice, again with restoration of lymphoid-specific defects and extension of the shortened lifespan, characteristic of ATM-deficient mice, and a reduced risk of lymphoid tumours. Delivery of these vectors to the brain represents an important challenge. James Dixon (University of Nottingham, Nottingham, UK) has described a cell-penetrating peptide-based nanoparticle system for delivery of ATM to the brain [80]. This approach is coupled with CRISPR gene editing technology to correct A-T radiosensitivity in patient cells and in the cerebellar region of mice. Masatoshi Takagi (Institute of Science, Tokyo, Japan) has recently described the construction of a helper-dependent adenoviral vector capable of genome integration using the PiggyBac transposon system [80]. He successfully transduced the *ATM* gene into an ATM-deficient cell line with a similar level of ATM expression to that observed endogenously, and efficient restoration of radiosensitivity. These are all encouraging approaches and render ATM delivery a promising therapy for the future.

### 5.2. Drug Screening and Repurposing for Restoration of ATM Function

Synthetic rescue platforms are being developed by INSMED to identify potential drug targets that are outside the disrupted biological pathway (https://insmed.com, (accessed on 6 August 2026. The rationale is that genes do not work in isolation but rather in networks that influence the activities of other genes and function in a broad range of diseases. This is evident from examples such as the earlier description of a genetic screen which revealed that inactivating genes encoding classical NHEJ proteins (LIG4, XRCC4, XLF) or those of the BRCA1-A complex alleviated the toxicity of TOP1 and PARP inhibitors in ATM-deficient cells by synthetic rescue [84]. In this case, ATM functions to prevent toxic DNA end-joining.

Rather than attempting to replace the defective ATM gene with a full-length normal copy, approaches have been pursued to enhance the DNA damage response in A-T cells. More recently, high-throughput screens of drug repurposing libraries consisting of approximately 6000 compounds to identify those that selectively upregulate the DNA damage response (damage-induced phosphorylation of CHK2) in A-T iPSCs [24]. Out of eight compounds identified, four showed theoretical potential from cellular thermal shift assays [85] in alleviating A-T neurological symptoms (Benzydiamine (anti-inflammatory); carbetapentane (antitussive); dicyclomine (anticholinergic) and deptropine (antihistamine).

### 5.3. UFMylation and ATM Activation

In response to DNA damage, ATM is activated by the MRE11 complex positioned at DNA DSBs involving both autophosphorylation and acetylation of the ATM protein [6,86]. More recently, it has been shown that Mre11 is UFMylated (ubiquitin-like modification), which is required for optimal ATM activation, homologous recombination DSB repair (HR) and genome integrity [87]. In a separate study, ATM activation was shown to involve UFMylation of histone H4 by UFL1 ligase following DNA damage and the subsequent recruitment of both Suv39h1 and Tip60 proteins [88]. This in turn leads to ATM phosphorylation of UFL1 E3 ligase activity, promoting ATM activation in a positive feedback loop. To add to the complexity, the STK38 kinase, which contains a potential UFM1-binding motif, recognises UFMylated H4 and recruits Suv39h1 to the DSB, resulting in H3K9 trimethylation and Tip60 activation to promote ATM activation [89]. In short, STK38 functions as a reader of monomethylated H4 at Lys31 to promote ATM activation. As expected, UFM1-specific peptidase 2 (UFSP2), which removes UFMylation, suppresses ATM activation. Under unperturbed conditions, UFSP2 binds the MRE11 complex, but in response to DNA DSB it is phosphorylated by ATM and dissociates from the complex. Phosphorylation of UFSP2 is removed by the phosphatase WIP1, which leads to its recruitment to the DNA DSB, deUFMylating H4 and suppressing ATM activation [90].

### 5.4. Modulating Glutamate

Metabolic profiling of A-T cells revealed altered fuel usage, showing that they become highly dependent on glutamine as an energy source for the TCA cycle. This vulnerability underlies a therapeutic strategy targeting the amino acid balance. Recently, it was shown that treatment with alpha-ketoglutarate (α-KG), the α-keto acid backbone of glutamine, demonstrated potential for alleviating glutamine dependence and Purkinje cell degeneration [91] Another recent study has illuminated the mechanism of action of N-acetyl cysteine (NAC), an antioxidant known to increase survival in Atm-null mice [92]. Research suggests the primary benefit of NAC may be related to metabolism, rather than solely reducing basal oxidative stress. Loss of ATM function impairs the phosphorylation of CD98HC, a chaperone protein critical for trafficking amino acid antiporters, specifically the cystine/glutamate antiporter (x_c_^−^). This defective trafficking impairs the function of the antiporters, leading to the accumulation of toxic levels of intracellular glutamate. NAC, which provides the rate-limiting substrate cysteine, allows the production of glutathione (GSH). This process bypasses the antiporter defect and reduces toxic glutamate accumulation, suggesting that NAC can rescue A-T phenotypes (such as glucose intolerance and fatty liver) by rebalancing intracellular glutamate pools. Glutamate is an important anaplerotic amino acid in the brain, which contributes to α-KG synthesis via glutamate-dehydrogenase and transaminases. α-KG feeds into the TCA to support ATP production, redox support, as well as biosynthesis of lipids, nucleotides and amino acids. This is critical under glucose metabolism stress conditions. Glutamate is not only a metabolic building block but is also a critical neurotransmitter, which is tightly controlled in the CNS. In the brain, astrocytes help regulate glutamate homeostasis via transporters (SLC1A2/SLC1A3), as well as converting glutamate to glutamine (via glutamine synthetase). Glutamate is transported across the inner mitochondrial membrane via SLC25A22 and SLC25A18, which help regulate anaplerotic flux into the TCA. Glutamate also generates NADH, feeds the malate-aspartate shuttle and supports NADPH production, important for ROS buffering and GSH synthesis. Importantly, uncontrolled glutamate in the CNS can drive excitotoxicity [93].

### 5.5. Read Through Compounds for Premature Stop Codons

Stop codons caused by nonsense mutations in the *ATM* gene account for approximately 30% of all mutations in A-T [94,95]. Thus, the use of compounds that induce the read-through of these premature termination codons (PTCs), giving rise to translation of full-length proteins, presents another potential approach to therapy in A-T. Compounds, including both aminoglycosides and non-aminoglycosides, have revealed read-through in several disease models including A-T [96]. High-throughput screening subsequently identified two novel derivative compounds, GJ071 and GJ072, which were effective in generating full-length ATM protein and improved cell survival after exposure of cells to ionising radiation [97]. These findings were extended to an animal model in a collaboration between Gatti and Paul Mathews [98]. They inserted null mutations in both the Atm(nonsense) and Pptx (knockout) genes in the same animal that was characterised by an ataxic phenotype associated with atrophy of the cerebellar molecular layer, including reduced membrane capacitance and lower action potential thresholds in Purkinje cells [98]. These mice also had impaired early thymocyte development and T-cell maturation and a high predisposition to developing thymus cancer. Use of the small molecule read-through (SMRT) compound GJ103 restored ATM production in both spleen and cerebellum. This mouse represents a valuable pre-clinical model for A-T for testing not only SMRT compounds but also other potential therapeutics such as antioxidants and compounds such as triheptanoin, NR, NAL and betamethasone.

### 5.6. ATM Substrates—Molecular Chaperones

A subgroup of molecular chaperones, the heat-shock proteins (HSPs), are involved in protein folding, assembly, stabilisation, and degradation to maintain cellular homeostasis in tissues including the nervous system. These proteins are also involved in the transformation of cells to cancerous forms [99]. Their role in combating neuroinflammation and protein aggregation is important in the progression of neurodegenerative diseases [100]. There is increasing evidence that HSPs and other chaperones contribute to the role of ATM in responding to oxidative and other forms of stress. Defective mitochondrial function and accumulation of ROS in ATM-deficient cells lead to oxidative DNA damage, Poly(ADP-ribose) polymerase (PARP) hyperactivation and eventually protein aggregation [101].

One of the responses to oxidative stress is the reconfiguring of carbohydrate metabolism from glycolysis into the pentose phosphate pathway (PPP), which is under the control of ATM. ATM promotes phosphorylation of HSP27, which induces its binding to glucose-6-phosphate dehydrogenase (G6PD), increasing the activity of G6PD and stimulating the PPP. These data point to a role for ATM in protecting cells against oxidative stress by stimulating NADPH production and promoting nucleotide synthesis for the repair of DNA damage [31].

The activity of a second member of this family, HSP90, is required for the stability of oncogenes including the receptor tyrosine kinase HER2 [31]. In a recent report, it was demonstrated that ATM induces the phosphorylation of HSP90β on T297, enhancing its ability to bind to and stabilise HER2 to promote its oncogenic potential [102]. The unphosphorylated mutant form A297 reduces its ability to interact with HER2, triggering ubiquitination and degradation of HER2 and, in this way, reducing the viability of HER2-overexpressing cells. Overall, these results point to ATM activation sustaining HER2 protein stability and driving tumourigenicity.

On the other hand, a recent report provides evidence that ATM phosphorylation of the HSP90 paralogue, GRP94, reduces its translocation to the plasma membrane to stabilise receptor tyrosine kinases such as HER2 and EGFR [103]. GRP94 is normally located on the ER, but under stressful conditions a change in N-glycosylation and protein conformation occurs, resulting in its translocation to the plasma membrane to function as a scaffold protein to stabilise these receptor molecules. Phosphorylation by ATM attenuated this translocation but in ATM-deficient cells increased levels of GRP94 and oncogenic receptors were observed, suggesting that this could account for the increased susceptibility to cancer in patients with A-T [103]. It is also of considerable interest that they demonstrated a similar mechanism operating in microglial activation, which has been shown to be defective in A-T [68,104,105,106]. Curtailing the level of cell surface GRP94 or inhibiting its activation by inhibitors such as PU-WS13 in A-T may represent an approach to treating patients with A-T.

The IP3R1-GRP75-VDAC1 (Figure 5) complex localises to the mitochondrial-associated membrane (MAM) and has been shown to play a major role in bridging between the ER and mitochondria, acting as an important control point of cell fate in conditions of oxidative and other forms of stress [107]. A component of this complex, GRP75, also an HSP paralogue, fails to assemble the complex in A-T cells in response to nutrient stress, resulting in defective Ca^2+^ release from the ER and uptake into the mitochondrion, leading to mitochondrial dysfunction [12]. Thus, it seems likely that ATM functions through this chaperone in controlling mitochondrial homeostasis since the triheptanoin metabolite heptanoate enhances ER-mitochondrial contacts, including the IP3R-GRP75-VDAC1 complex, in ATM-deficient cells [55]. As outlined above, this has been the basis for a clinical trial with A-T patients [56].

### 5.7. Employing Model Systems to Study the Cellular Phenotype in A-T

A number of models have been generated for A-T, but only the recent description of a macaque model recapitulates the progressive cerebellar atrophy and accompanying Purkinje cell loss [23]. While several Atm-deficient mouse models have been described that exhibit key elements of the phenotype described in patients with A-T, they do not show the progressive neurodegeneration [23]. They exhibit a range of neurological abnormalities, including reduced performance in behavioural tests, cerebellar pathology, neuronal death in in vitro cultures, reduced synchronisation persistence in neural networks, vascular abnormalities and degeneration of the nigro-striatal and other pathways [108,109,110,111,112]. While no Purkinje cell defects were apparent, in situ survival of cultured cerebellar Purkinje cells was significantly shorter for Atm−/− than for Atm+/+ mice, but the inclusion of a nitroxide antioxidant CTMIO prevented Purkinje cell death and enhanced dendritogenesis [111]. On the other hand, a rat model did not display cerebellar atrophy but was instead characterised by paralysis and spinal cord atrophy [104]. These animals had a significant loss of motor neurons and microgliosis of the spinal cord. Bethamethasone extended the lifespan of the Atm−/− rats, prevented microglial activation and significantly reduced neuroinflammatory changes and motor neuron loss. This aberrant activation of microglia had been observed previously in cerebellar tissue from five patients with A-T [113] and appears to contribute significantly to the neurodegenerative phenotype [106]. In a compelling study, single-nuclei RNA sequencing of A-T post-mortem cerebella demonstrated microglial activation, reasoned to be cell-intrinsic and driving death of cerebellar neurons. Microglia were the cell type with the highest ATM expression in the brain, and exhibited activated, pro-inflammatory gene programs. This was more pronounced in the cerebellum compared to the cortex [68]. The concept of cell-intrinsic activation upstream of neuronal cell death presents an interesting opportunity that targeting upstream processes might prove a potent strategy for modulating the neurodegenerative aspect, as recently demonstrated in Friedrich’s Ataxia [114].

Further support for glial cell involvement comes from *Drosophila melanogaster* ATM mutants where Relish (NF-*k*B superfamily member) activation in glial cells is sufficient to cause neurodegeneration [115]. Cerebellar lesions were also reported in a pig model of A-T but no evidence of neurodegeneration [116]. The A-T model with closest similarity to the human syndrome is the recently published macaque model, which exhibits the major hallmark features including growth retardation, lymphopenia, elevated α-fetoprotein, oculocutaneous telangiectasia, radiation hypersensitivity, cerebellar atrophy, Purkinje cell loss and cerebellar degeneration [23]. The latter model represents a valuable tool to understand the neuropathology in A-T and an important resource for the further development of therapeutic strategies.

### 5.8. In Vitro Model Systems of the CNS for A-T

Induced pluripotent stem cells (iPSCs) derived from patients with A-T have now been generated from multiple groups [117,118,119,120], allowing a limitless supply of cells which are capable of giving rise to all germ lineages. One of the major utilities of this cell type is patterning to produce neuronal cells of the cerebellum, either as 2D monolayers or self-organising 3D organoids [121,122,123]. Cerebellar organoids have emerged as powerful in vitro models for studying human cerebellar development and disease, bridging a critical gap between animal models and patient tissue. These 3D cultures recapitulate key aspects of cerebellar architecture, including the generation of Purkinje-like neurons, granule cells, and supporting glial populations, as well as the temporal progression of maturation programs, albeit at early developmental stages. These cell culture systems offer a tool to help understand the developmental nature of A-T, provide the means to perform large-scale drug screening activity, and may represent the basis for the future development of regenerative therapies.

### 5.9. Clinical Progress with Other Ataxias

In looking at progress being made amongst the broader group of other inherited ataxias, therapeutic strategies have focused on strategies modifying disease, as well as symptomatic pharmacotherapies. Like A-T, oxidative stress and mitochondrial dysfunction are characteristic of Friedreich’s ataxia (FRDA, OMIM 229300), which has spurred the development and recent FDA approval of the NRF2 activator omaveloxolone (Skyclarys). Omaveloxolone significantly improved neurological function compared with placebo [124] in the multicentre MOXIe randomised trial and has been reviewed as the first approved ataxia-specific drug [125,126]. Gene and protein-targeted strategies to increase frataxin expression, including epigenetic modulation, gene replacement, and iron chelation/antioxidants, are under active investigation (e.g., interferon gamma, nicotinamide, CoQ10/idebenone). For spinocerebellar ataxias (SCAs), symptomatic trials have explored riluzole, which demonstrated some reduction in ataxia severity scores in small double-blind studies [127,128], and other repurposed drugs, although consistent disease-modifying effects remain to be established. Additionally, trials of ASOs and RNA-targeting approaches are in early phases for polyglutamine SCAs, reflecting a precision-medicine direction for therapy [129].

### 5.10. Combination Therapy for A-T

While ASOs and other gene therapies may be applicable for specific A-T patients, there is an urgent need to develop other therapies. Given the multisystemic and progressive nature of A-T, it is likely that combinatorial therapies will be necessary, spanning across organ systems and disciplines described above. One example of this is the potential combination of synergistic agents such as C7 and NR, which individually address deficits in specific branches of central carbon metabolism. In support of this approach, a recent large-scale drug screen in iPSC-derived motor neurons from ALS patients showed that screening of 97% of drugs previously tested in ALS clinical trials failed. However, the combinatorial testing of drugs identified that baricitinib, memantine and riluzole effectively promoted survival through their combined action [130].

## 6. Conclusions

At present, there is no cure for A-T, but advances in gene technology and some results from clinical trials provide evidence for the development of effective therapy for patients in the near future. These trials are varied in their design and focus largely on correcting mitochondrial function (Triheptanoin, NR) or the inflammatory phenotype, which are thought to be significant factors in the progression of the disease. While these trials may not have yet provided a cure for A-T patients, they have already made significant contributions to quality of life, with improvements to balance and coordination, improvements to speech, increasing energy levels as well as self-confidence and greater independence.

## Data Availability

No new data were created or analysed in this study. Data sharing is not applicable to this article.

## References

[1] Savitsky K., Bar-Shira A., Gilad S., Rotman G., Ziv Y., Vanagaite L., A Tagle D., Smith S., Uziel T., Sfez S. (1995). A Single Ataxia Telangiectasia Gene with a Product Similar to PI-3 Kinase. Science.

[2] Lavin M.F. (2008). Ataxia-telangiectasia: From a rare disorder to a paradigm for cell signalling and cancer. Nat. Rev. Mol. Cell Biol..

[3] McKinnon P.J. (2012). ATM and the Molecular Pathogenesis of Ataxia Telangiectasia. Annu. Rev. Pathol. Mech. Dis..

[4] Veenhuis S., van Os N., Weemaes C., Kamsteeg E.J., Willemsen M., Adam M.P., Bick S., Mirzaa G.M., Pagon R.A., Wallace S.E., Amemiya A. (1999). Ataxia-Telangiectasia. GeneReviews®.

[5] Shiloh Y., Ziv Y. (2013). The ATM protein kinase: Regulating the cellular response to genotoxic stress, and more. Nat. Rev. Mol. Cell Biol..

[6] Bakkenist C.J., Kastan M.B. (2003). DNA damage activates ATM through intermolecular autophosphorylation and dimer dissociation. Nature.

[7] Uziel T., Lerenthal Y., Moyal L., Andegeko Y., Mittelman L., Shiloh Y. (2003). Requirement of the MRN complex for ATM activation by DNA damage. EMBO J..

[8] Paull T.T., Woolley P.R. (2024). A-T neurodegeneration and DNA damage-induced transcriptional stress. DNA Repair.

[9] Guo Z., Kozlov S., Lavin M.F., Person M.D., Paull T.T. (2010). ATM activation by oxidative stress. Science.

[10] Andreyev A.Y., Kushnareva Y.E., Starkov A.A. (2005). Mitochondrial metabolism of reactive oxygen species. Biochemistry.

[11] Howes A.C., Perisic O., Williams R.L. (2023). Structural insights into the activation of ataxia-telangiectasia mutated by oxidative stress. Sci. Adv..

[12] Yeo A.J., Chong K.L., Gatei M., Zou D., Stewart R., Withey S., Wolvetang E., Parton R.G., Brown A.D., Kastan M.B. (2021). Impaired endoplasmic reticulum-mitochondrial signaling in ataxia-telangiectasia. iScience.

[13] Bastianello G., Porcella G., Beznoussenko G.V., Kidiyoor G., Ascione F., Li Q., Cattaneo A., Matafora V., Disanza A., Quarto M. (2023). Cell stretching activates an ATM mechano-transduction pathway that remodels cytoskeleton and chromatin. Cell Rep..

[14] Crawford T.O., Skolasky R.L., Fernandez R., Rosquist K.J., Lederman H.M. (2005). Survival probability in ataxia telangiectasia. Arch. Dis. Child..

[15] Nowak-Wegrzyn A., Crawford T.O., Winkelstein J.A., Carson K.A., Lederman H.M. (2004). Immunodeficiency and infections in ataxia-telangiectasia. J. Pediatr..

[16] Driessen G.J., Ijspeert H., Weemaes C.M., Haraldsson Á., Trip M., Warris A., van der Flier M., Wulffraat N., Verhagen M.M., Taylor M.A. (2013). Antibody deficiency in patients with ataxia telangiectasia is caused by disturbed B- and T-cell homeostasis and reduced immune repertoire diversity. J. Allergy Clin. Immunol..

[17] Farr A.K., Shalev B., Crawford T.O., Lederman H.M., Winkelstein J.A., Repka M.X. (2002). Ocular manifestations of ataxia-telangiectasia. Am. J. Ophthalmol..

[18] Lefton-Greif M.A., Crawford T.O., Winkelstein J.A., Loughlin G.M., Koerner C.B., Zahurak M., Lederman H.M. (2000). Oropharyngeal dysphagia and aspiration in patients with ataxia-telangiectasia. J. Pediatr..

[19] Weiss B., Krauthammer A., Soudack M., Lahad A., Sarouk I., Somech R., Heimer G., Ben-Zeev B., Nissenkorn A. (2016). Liver Disease in Pediatric Patients With Ataxia Telangiectasia: A Novel Report. J. Pediatr. Gastroenterol. Nutr..

[20] Donath H., Woelke S., Theis M., Heß U., Knop V., Herrmann E., Krauskopf D., Kieslich M., Schubert R., Zielen S. (2019). Progressive Liver Disease in Patients With Ataxia Telangiectasia. Front. Pediatr..

[21] Rothblum-Oviatt C., Wright J., Lefton-Greif M.A., McGrath-Morrow S.A., Crawford T.O., Lederman H.M. (2016). Ataxia telangiectasia: A review. Orphanet J. Rare Dis..

[22] Woelke S., Valesky E., Bakhtiar S., Pommerening H., Pfeffermann L.M., Schubert R., Zielen S. (2018). Treatment of Granulomas in Patients With Ataxia Telangiectasia. Front. Immunol..

[23] Xu K., Zhang Y., Chen Y., Zhu X., Li Y., Lv L., He X., Hu Z., Li Y., Ye M. (2025). ATM deficiency drives phenotypic diversity and Purkinje cell degeneration in a macaque model of ataxia-telangiectasia. Cell Rep. Med..

[24] Jayanth N., Mahé G., Campbell M., Lipkin M., Jain S., van de Bospoort R., Thornton J., Margus B., Fischer D.F. (2024). Drug repurposing screen for the rare disease ataxia-telangiectasia. SLAS Discov. Adv. Sci. Drug Discov..

[25] Chen S., Heendeniya S.N., Le B.T., Rahimizadeh K., Rabiee N., Zahra Q.U.A., Veedu R.N. (2024). Splice-Modulating Antisense Oligonucleotides as Therapeutics for Inherited Metabolic Diseases. BioDrugs.

[26] Kim J., Woo S., de Gusmao C.M., Zhao B., Chin D.H., DiDonato R.L., Nguyen M.A., Nakayama T., Hu C.A., Soucy A. (2023). A framework for individualized splice-switching oligonucleotide therapy. Nature.

[27] Wilton-Clark H., Yan E., Yokota T. (2024). Preparing for Patient-Customized N-of-1 Antisense Oligonucleotide Therapy to Treat Rare Diseases. Genes.

[28] Howarth C., Gleeson P., Attwell D. (2012). Updated Energy Budgets for Neural Computation in the Neocortex and Cerebellum. J. Cereb. Blood Flow Metab..

[29] Chow H.-M., Cheng A., Song X., Swerdel M.R., Hart R.P., Herrup K. (2019). ATM is activated by ATP depletion and modulates mitochondrial function through NRF1. J. Cell Biol..

[30] Westi E.W., Carlsen R.B., Andersen J.V., Kilic Z., Alnajar D., Holmgaard N.K., Sintes B.G., Cota-Coronado A., Muñoz S.S., Galvagnion C. (2025). Metabolic Flexibility of Microglia: Energy Substrate Utilization and Impact on Neuronal Metabolism. J. Neurochem..

[31] Cosentino C., Grieco D., Costanzo V. (2010). ATM activates the pentose phosphate pathway promoting anti-oxidant defence and DNA repair. EMBO J..

[32] Stern N., Hochman A., Zemach N., Weizman N., Hammel I., Shiloh Y., Rotman G., Barzilai A. (2002). Accumulation of DNA Damage and Reduced Levels of Nicotine Adenine Dinucleotide in the Brains of Atm-deficient Mice. J. Biol. Chem..

[33] Ambrose M., Goldstine J.V., Gatti R.A. (2007). Intrinsic mitochondrial dysfunction in ATM-deficient lymphoblastoid cells. Hum. Mol. Genet..

[34] Eaton J.S., Lin Z.P., Sartorelli A.C., Bonawitz N.D., Shadel G.S. (2007). Ataxia-telangiectasia mutated kinase regulates ribonucleotide reductase and mitochondrial homeostasis. J. Clin. Investig..

[35] Mercer J.R., Yu E., Figg N., Cheng K.-K., Prime T.A., Griffin J.L., Masoodi M., Vidal-Puig A., Murphy M.P., Bennett M.R. (2012). The mitochondria-targeted antioxidant MitoQ decreases features of the metabolic syndrome in ATM+/–/ApoE–/– mice. Free Radic. Biol. Med..

[36] D’Souza A.D., A Parish I., Krause D.S., Kaech S.M., Shadel G.S. (2013). Reducing Mitochondrial ROS Improves Disease-related Pathology in a Mouse Model of Ataxia-telangiectasia. Mol. Ther..

[37] Sharma N.K., Lebedeva M., Thomas T., Kovalenko O.A., Stumpf J.D., Shadel G.S., Santos J.H. (2014). Intrinsic mitochondrial DNA repair defects in Ataxia Telangiectasia. DNA Repair.

[38] Blignaut M., Loos B., Botchway S.W., Parker A.W., Huisamen B. (2019). Ataxia-Telangiectasia Mutated is located in cardiac mitochondria and impacts oxidative phosphorylation. Sci. Rep..

[39] Morita A., Tanimoto K., Murakami T., Morinaga T., Hosoi Y. (2014). Mitochondria are required for ATM activation by extranuclear oxidative stress in cultured human hepatoblastoma cell line Hep G2 cells. Biochem. Biophys. Res. Commun..

[40] Kamsler A., Daily D., Hochman A., Stern N., Shiloh Y., Rotman G., Barzilai A. (2001). Increased oxidative stress in ataxia telangiectasia evidenced by alterations in redox state of brains from Atm-deficient mice. Cancer Res..

[41] Chen P., Peng C., Luff J., Spring K., Watters D., Bottle S., Furuya S., Lavin M.F. (2003). Oxidative Stress Is Responsible for Deficient Survival and Dendritogenesis in Purkinje Neurons from Ataxia-Telangiectasia Mutated Mutant Mice. J. Neurosci..

[42] Reliene R., Fischer E., Schiestl R.H. (2004). Effect of *N*-Acetyl Cysteine on Oxidative DNA Damage and the Frequency of DNA Deletions in *Atm*-Deficient Mice. Cancer Res..

[43] Fang E.F., Kassahun H., Croteau D.L., Scheibye-Knudsen M., Marosi K., Lu H., Shamanna R.A., Kalyanasundaram S., Bollineni R.C., Wilson M.A. (2016). NAD + Replenishment Improves Lifespan and Healthspan in Ataxia Telangiectasia Models via Mitophagy and DNA Repair. Cell Metab..

[44] Yang B., Dan X., Hou Y., Lee J., Wechter N., Krishnamurthy S., Kimura R., Babbar M., Demarest T., McDevitt R. (2021). NAD+ supplementation prevents STING-induced senescence in ataxia telangiectasia by improving mitophagy. Aging Cell.

[45] Zong L., Tanaka-Yano M., Park B., Yanai H., Turhan F.T., Croteau D.L., Tian J., Fang E.F., Bohr V.A., Beerman I. (2021). NAD+ augmentation with nicotinamide riboside improves lymphoid potential of Atm−/− and old mice HSCs. npj Aging Mech. Dis..

[46] Haj M., Frey Y., Levon A., Maliah A., Ben-Yishay T., Slutsky R., Smoom R., Tzfati Y., Ben-David U., Levy C. (2025). The cGAS–STING, p38 MAPK, and p53 pathways link genome instability to accelerated cellular senescence in ATM-deficient murine lung fibroblasts. Proc. Natl. Acad. Sci. USA.

[47] Yusri K., Jose S., Vermeulen K.S., Tan T.C.M., Sorrentino V. (2025). The role of NAD+ metabolism and its modulation of mitochondria in aging and disease. npj Metab. Health Dis..

[48] Veenhuis S.J., van Os N.J., Janssen A.J., van Gerven M.H., Coene K.L., Engelke U.F., Wevers R.A., Tinnevelt G.H., ter Heine R., van de Warrenburg B.P. (2021). Nicotinamide Riboside Improves Ataxia Scores and Immunoglobulin Levels in Ataxia Telangiectasia. Mov. Disord..

[49] Presterud R., Deng W.H., Wennerström A.B., Burgers T., Gajera B., Mattsson K., Solberg A., Fang E.F., Nieminen A.I., Stray-Pedersen A. (2023). Long-Term Nicotinamide Riboside Use Improves Coordination and Eye Movements in Ataxia Telangiectasia. Mov. Disord..

[50] Donath H., Woelke S., Schubert R., Kieslich M., Theis M., Auburger G., Duecker R.P., Zielen S. (2022). Neurofilament Light Chain is a Biomarker of Neurodegeneration in Ataxia Telangiectasia. Cerebellum.

[51] Steinbruecker K., Tiefenthaler E., Schernthaner E.-M., Jungwirth J., Wortmann S.B. (2022). Nicotinamide Riboside for Ataxia Telangiectasia: A Report of an Early Treated Individual. Neuropediatrics.

[52] Vockley J. (2020). Long-chain fatty acid oxidation disorders and current management strategies. Am. J. Manag. Care.

[53] Sklirou E., Alodaib A.N., Dobrowolski S.F., Mohsen A.-W.A., Vockley J. (2021). Physiological Perspectives on the Use of Triheptanoin as Anaplerotic Therapy for Long Chain Fatty Acid Oxidation Disorders. Front. Genet..

[54] Vockley J., Burton B.K., Berry G., Longo N., Phillips J., Sanchez-Valle A., Chapman K.A., Tanpaiboon P., Grunewald S., Murphy E. (2023). Triheptanoin for the treatment of long-chain fatty acid oxidation disorders: Final results of an open-label, long-term extension study. J. Inherit. Metab. Dis..

[55] Yeo A., Subramanian G., Chong K., Gatei M., Parton R., Coman D., Lavin M. (2021). An anaplerotic approach to correct the mitochondrial dysfunction in ataxia-telangiectasia (A-T). Mol. Metab..

[56] Lynch M., Manoy S., Sly P.D., Wainwright C.E., Wolvetang E., Feenstra J.E., Dowling J., Ware R.S., Patel M.S., Hermith-Ramirez D. (2025). Phase 2a/b randomised placebo-controlled dose-escalation trial of triheptanoin for ataxia-telangiectasia: Treating mitochondrial dysfunction with anaplerosis. EBioMedicine.

[57] Kaya E., Smith D.A., Smith C., Boland B., Strupp M., Platt F.M. (2020). Beneficial Effects of Acetyl-DL-Leucine (ADLL) in a Mouse Model of Sandhoff Disease. J. Clin. Med..

[58] Vibert N., Vidal P. (2001). In vitro effects of acetyl-dl-leucine (tanganil®) on central vestibular neurons and vestibulo-ocular networks of the guinea-pig. Eur. J. Neurosci..

[59] Hegdekar N., Lipinski M.M., Sarkar C. (2021). N-Acetyl-l-leucine improves functional recovery and attenuates cortical cell death and neuroinflammation after traumatic brain injury in mice. Sci. Rep..

[60] Sarkar C., Lipinski M.M. (2023). Autophagy in neuroinflammation after traumatic brain injury. Neural Regen. Res..

[61] Kaya E., A Smith D., Smith C., Morris L., Bremova-Ertl T., Cortina-Borja M., Fineran P., Morten K.J., Poulton J., Boland B. (2020). Acetyl-leucine slows disease progression in lysosomal storage disorders. Brain Commun..

[62] Churchill G.C., Strupp M., Factor C., Bremova-Ertl T., Factor M., Patterson M.C., Platt F.M., Galione A. (2021). Acetylation turns leucine into a drug by membrane transporter switching. Sci. Rep..

[63] Brueggemann A., Bicvic A., Goeldlin M., Kalla R., Kerkeni H., Mantokoudis G., Abegg M., Kolníková M., Mohaupt M., Bremova-Ertl T. (2021). Effects of Acetyl-DL-Leucine on Ataxia and Downbeat-Nystagmus in Six Patients With Ataxia Telangiectasia. J. Child Neurol..

[64] Saberi-Karimian M., Houra M., Jamialahmadi T., Sarvghadi P., Nikbaf M., Akhlaghi S., Sahebkar A. (2022). The Effects of N-Acetyl-L-Leucine on the Improvement of Symptoms in a Patient with Multiple Sulfatase Deficiency. Cerebellum.

[65] Beyraghi-Tousi M., Sahebkar A., Houra M., Sarvghadi P., Jamialahmadi T., Bagheri R., Tavallaie S., Gumpricht E., Saberi-Karimian M. (2024). Efficacy and safety of N-acetyl-L-leucine in patients with ataxia telangiectasia: A randomized, double-blind, placebo-controlled, crossover clinical trial. Eur. J. Paediatr. Neurol..

[66] Ehrlich L.A., Yang-Iott K., DeMicco A., Bassing C.H. (2015). Somatic inactivation of ATM in hematopoietic cells predisposes mice to cyclin D3 dependent T cell acute lymphoblastic leukemia. Cell Cycle.

[67] A McGrath-Morrow S., Rothblum-Oviatt C.C., Wright J., Schlechter H., A Lefton-Greif M., A Natale V., O Crawford T., Lederman H.M. (2021). Multidisciplinary Management of Ataxia Telangiectasia: Current Perspectives. J. Multidiscip. Heal..

[68] Lai J., Demirbas D., Kim J., Jeffries A.M., Tolles A., Park J., Chittenden T.W., Buckley P.G., Yu T.W., Lodato M.A. (2023). ATM-deficiency-induced microglial activation promotes neurodegeneration in ataxia-telangiectasia. Cell Rep..

[69] McGrath-Morrow S.A., Collaco J.M., Crawford T.O., Carson K.A., Lefton-Greif M.A., Zeitlin P., Lederman H.M. (2010). Elevated Serum IL-8 Levels in Ataxia Telangiectasia. J. Pediatr..

[70] McGrath-Morrow S.A., Collaco J.M., Detrick B., Lederman H.M. (2016). Serum Interleukin-6 Levels and Pulmonary Function in Ataxia-Telangiectasia. J. Pediatr..

[71] Biagiotti S., Bianchi M., Rossi L., Chessa L., Magnani M. (2019). Activation of NRF2 by dexamethasone in ataxia telangiectasia cells involves KEAP1 inhibition but not the inhibition of p38. PLoS ONE.

[72] Biagiotti S., Menotta M., Orazi S., Spapperi C., Brundu S., Fraternale A., Bianchi M., Rossi L., Chessa L., Magnani M. (2016). Dexamethasone improves redox state in ataxia telangiectasia cells by promoting an NRF2-mediated antioxidant response. FEBS J..

[73] Menotta M., Biagiotti S., Bianchi M., Chessa L., Magnani M. (2012). Dexamethasone Partially Rescues Ataxia Telangiectasia-mutated (ATM) Deficiency in Ataxia Telangiectasia by Promoting a Shortened Protein Variant Retaining Kinase Activity. J. Biol. Chem..

[74] Biagiotti S., Menotta M., Giacomini E., Radici L., Bianchi M., Bozzao C., Chessa L., Magnani M. (2014). Forward subtractive libraries containing genes transactivated by dexamethasone in ataxia-telangiectasia lymphoblastoid cells. Mol. Cell. Biochem..

[75] Giardino G., Fusco A., Romano R., Gallo V., Maio F., Esposito T., Palamaro L., Parenti G., Salerno M.C., Vajro P. (2012). Betamethasone therapy in ataxia telangiectasia: Unraveling the rationale of this serendipitous observation on the basis of the pathogenesis. Eur. J. Neurol..

[76] Zannolli R., Buoni S., Betti G., Salvucci S., Plebani A., Soresina A., Pietrogrande M.C., Martino S., Leuzzi V., Finocchi A. (2012). A randomized trial of oral betamethasone to reduce ataxia symptoms in ataxia telangiectasia. Mov. Disord..

[77] Chessa L., Leuzzi V., Plebani A., Soresina A., Micheli R., D’aGnano D., Venturi T., Molinaro A., Fazzi E., Marini M. (2014). Intra-Erythrocyte Infusion of Dexamethasone Reduces Neurological Symptoms in Ataxia Teleangiectasia Patients: Results of a Phase 2 Trial. Orphanet J. Rare Dis..

[78] Leuzzi V., Micheli R., D’AGnano D., Molinaro A., Venturi T., Plebani A., Soresina A., Marini M., Leali P.F., Quinti I. (2015). Positive effect of erythrocyte-delivered dexamethasone in ataxia-telangiectasia. Neurol.-Neuroimmunol. Neuroinflamm..

[79] Ozdin D., Kheibarshekan L., Mambrini G., Tremblay P. (2025). Population Pharmacokinetic Modeling and Pediatric Exposure of Dexamethasone Sodium Phosphate Encapsulated in Erythrocytes (eDSP) Administered Monthly for Treatment of Neurological Symptoms of Patients With Ataxia Telangiectasia. CPT Pharmacomet. Syst. Pharmacol..

[80] Coman D., Jeggo P., Lavin M. (2025). Lets talk about ataxia-telangiectasia: Meeting report of the AT clinical research conference June 2025. DNA Repair.

[81] Taylor A., Lam Z., Last J., Byrd P. (2014). Ataxia telangiectasia: More variation at clinical and cellular levels. Clin. Genet..

[82] Zhang N., Chen P., Khanna K.K., Scott S., Gatei M., Kozlov S., Watters D., Spring K., Yen T., Lavin M.F. (1997). Isolation of full-length ATM cDNA and correction of the ataxia-telangiectasia cellular phenotype. Proc. Natl. Acad. Sci. USA.

[83] A Ovchinnikov D., Withey S.L., Leeson H.C., Lei U.W., Sundarrajan A., Junday K., Pewarchuk M., Yeo A.J., Kijas A.W., Lavin M.F. (2020). Correction of ATM mutations in iPS cells from two ataxia-telangiectasia patients restores DNA damage and oxidative stress responses. Hum. Mol. Genet..

[84] Balmus G., Pilger D., Coates J., Demir M., Sczaniecka-Clift M., Barros A.C., Woods M., Fu B., Yang F., Chen E. (2019). ATM orchestrates the DNA-damage response to counter toxic non-homologous end-joining at broken replication forks. Nat. Commun..

[85] Jafari R., Almqvist H., Axelsson H., Ignatushchenko M., Lundbäck T., Nordlund P., Molina D.M. (2014). The cellular thermal shift assay for evaluating drug target interactions in cells. Nat. Protoc..

[86] Sun Y., Jiang X., Chen S., Fernandes N., Price B.D. (2005). A role for the Tip60 histone acetyltransferase in the acetylation and activation of ATM. Proc. Natl. Acad. Sci. USA.

[87] Wang Z., Gong Y., Peng B., Shi R., Fan D., Zhao H., Zhu M., Zhang H., Lou Z., Zhou J. (2019). MRE11 UFMylation promotes ATM activation. Nucleic Acids Res..

[88] Qin B., Yu J., Nowsheen S., Wang M., Tu X., Liu T., Li H., Wang L., Lou Z. (2019). UFL1 promotes histone H4 ufmylation and ATM activation. Nat. Commun..

[89] Qin B., Yu J., Nowsheen S., Zhao F., Wang L., Lou Z. (2020). STK38 promotes ATM activation by acting as a reader of histone H4 ufmylation. Sci. Adv..

[90] Qin B., Yu J., Zhao F., Huang J., Zhou Q., Lou Z. (2022). Dynamic recruitment of UFM1-specific peptidase 2 to the DNA double-strand breaks regulated by WIP1. Genome Instab. Dis..

[91] Sun J.K.-L., Hart R.P., Herrup K., Peng A.Z., Wong G.C.-N., Wu D., Kwan K.-M., Chow K.H.-M. (2025). Alpha-ketoglutarate mitigates insulin resistance and metabolic inflexibility in a mouse model of Ataxia-Telangiectasia. Nat. Commun..

[92] Romero J.C., Tonapi S.S., Parihar M., Loranc E., Miller H.E., Lawrence L.A., Bassani N., Robledo D.G., Cao L., Nie J. (2025). Loss of CD98HC phosphorylation by ATM impairs antiporter trafficking and drives glutamate toxicity in Ataxia telangiectasia. Nat. Commun..

[93] McKenna M.C. (2007). The glutamate-glutamine cycle is not stoichiometric: Fates of glutamate in brain. J. Neurosci. Res..

[94] Wright J., Teraoka S., Onengut S., Tolun A., A Gatti R., Ochs H.D., Concannon P. (1996). A high frequency of distinct ATM gene mutations in ataxia-telangiectasia. Am. J. Hum. Genet..

[95] Micol R., Ben Slama L., Suarez F., Le Mignot L., Beauté J., Mahlaoui N., Dubois d’Enghien C., Laugé A., Hall J., Couturier J. (2011). Morbidity and mortality from ataxia-telangiectasia are associated with ATM genotype. J. Allergy Clin. Immunol..

[96] Lai C.-H., Chun H.H., Nahas S.A., Mitui M., Gamo K.M., Du L., Gatti R.A. (2004). Correction of ATM gene function by aminoglycoside-induced read-through of premature termination codons. Proc. Natl. Acad. Sci. USA.

[97] Du L., E Jung M., Damoiseaux R., Completo G., Fike F., Ku J.-M., Nahas S., Piao C., Hu H., A Gatti R. (2013). A New Series of Small Molecular Weight Compounds Induce Read Through of All Three Types of Nonsense Mutations in the ATM Gene. Mol. Ther..

[98] Perez H., Abdallah M.F., I Chavira J., Norris A.S., Egeland M.T., Vo K.L., Buechsenschuetz C.L., Sanghez V., Kim J.L., Pind M. (2021). A novel, ataxic mouse model of ataxia telangiectasia caused by a clinically relevant nonsense mutation. eLife.

[99] Mahalingam D., Swords R., Carew J.S., Nawrocki S.T., Bhalla K., Giles F.J. (2009). Targeting HSP90 for cancer therapy. Br. J. Cancer.

[100] Saxena S.K., Sharma D., Kumar S., Maurya V.K., Ansari S., Malhotra H.S., Singh A. (2025). Decoding the role of large heat shock proteins in the progression of neuroinflammation-mediated neurodegenerative disorders. Neuroprotection.

[101] Lee J.-H. (2024). Oxidative stress and the multifaceted roles of ATM in maintaining cellular redox homeostasis. Redox Biol..

[102] Fianco G., Taddei I., Oropallo V., Contadini C., Ferri A., Coculo L., Serapian S.A., Colombo G., Stagni V., Barilà D. (2026). ATM kinase phosphorylates HSP90 on T297 changing its conformation dynamics and promoting its interaction with HER2 receptor tyrosine kinase. Biochim. Biophys. Acta (BBA)-Mol. Cell Res..

[103] Burrell P.E., Fleenor D.E., Nicholson O.M., Shao C., Kastan M.B. (2025). ATM interaction with GRP94 modulates oncogenic receptor expression and signaling and microglial activation. Proc. Natl. Acad. Sci. USA.

[104] Quek H., Luff J., Cheung K., Kozlov S., Gatei M., Lee C.S., Bellingham M.C., Noakes P.G., Lim Y.C., Barnett N.L. (2016). A rat model of ataxia-telangiectasia: Evidence for a neurodegenerative phenotype. Hum. Mol. Genet..

[105] Bourseguin J., Cheng W., Talbot E., Hardy L., Lai J., Jeffries A.M., A Lodato M., Lee E.A., Khoronenkova S.V. (2022). Persistent DNA damage associated with ATM kinase deficiency promotes microglial dysfunction. Nucleic Acids Res..

[106] Levi H., Bar E., Cohen-Adiv S., Sweitat S., Kanner S., Galron R., Mitiagin Y., Barzilai A. (2021). Dysfunction of cerebellar microglia in Ataxia-telangiectasia. Glia.

[107] Honrath B., Metz I., Bendridi N., Rieusset J., Culmsee C., Dolga A.M. (2017). Glucose-regulated protein 75 determines ER–mitochondrial coupling and sensitivity to oxidative stress in neuronal cells. Cell Death Discov..

[108] Kuljis R.O., Xu Y., Aguila M.C., Baltimore D. (1997). Degeneration of neurons, synapses, and neuropil and glial activation in a murine *Atm* knockout model of ataxia–telangiectasia. Proc. Natl. Acad. Sci. USA.

[109] Eilam R., Peter Y., Elson A., Rotman G., Shiloh Y., Groner Y., Segal M. (1998). Selective loss of dopaminergic nigro-striatal neurons in brains of Atm-deficient mice. Proc. Natl. Acad. Sci. USA.

[110] Borghesani P.R., Alt F.W., Bottaro A., Davidson L., Aksoy S., Rathbun G.A., Roberts T.M., Swat W., Segal R.A., Gu Y. (2000). Abnormal development of Purkinje cells and lymphocytes in *Atm* mutant mice. Proc. Natl. Acad. Sci. USA.

[111] Gueven N., Luff J., Peng C., Hosokawa K., Bottle S.E., Lavin M.F. (2006). Dramatic extension of tumor latency and correction of neurobehavioral phenotype in Atm-mutant mice with a nitroxide antioxidant. Free Radic. Biol. Med..

[112] Raz-Prag D., Galron R., Segev-Amzaleg N., Solomon A.S., Shiloh Y., Barzilai A., Frenkel D. (2011). A Role for Vascular Deficiency in Retinal Pathology in a Mouse Model of Ataxia-Telangiectasia. Am. J. Pathol..

[113] Bottini A.R., Gatti R.A., Wirenfeldt M., Vinters H.V. (2011). Heterotopic Purkinje cells in ataxia-telangiectasia. Neuropathology.

[114] Pernaci C., Johnson A., Gillette S., Warden A.S., McCormick C., Weiser-Novak S., Ramirez G., Broersma E.H., Mishra P., Sivakumar A. (2026). Microgliopathy as a primary mediator of neuronal death in models of Friedreich’s Ataxia. Nat. Commun..

[115] Petersen A.J., Katzenberger R.J., A Wassarman D. (2013). The Innate Immune Response Transcription Factor Relish Is Necessary for Neurodegeneration in a *Drosophila* Model of Ataxia-Telangiectasia. Genetics.

[116] Beraldi R., Chan C.-H., Rogers C.S., Kovács A.D., Meyerholz D.K., Trantzas C., Lambertz A.M., Darbro B.W., Weber K.L., White K.A. (2015). A novel porcine model of ataxia telangiectasia reproduces neurological features and motor deficits of human disease. Hum. Mol. Genet..

[117] Nayler S., Gatei M., Kozlov S., Gatti R., Mar J.C., Wells C.A., Lavin M., Wolvetang E. (2012). Induced Pluripotent Stem Cells from Ataxia-Telangiectasia Recapitulate the Cellular Phenotype. STEM CELLS Transl. Med..

[118] Fukawatase Y., Toyoda M., Okamura K., Nakamura K.-I., Nakabayashi K., Takada S., Yamazaki-Inoue M., Masuda A., Nasu M., Hata K. (2014). Ataxia telangiectasia derived iPS cells show preserved x-ray sensitivity and decreased chromosomal instability. Sci. Rep..

[119] Bhatt N., Ghosh R., Roy S., Gao Y., Armanios M., Cheng L., Franco S. (2016). Robust reprogramming of Ataxia-Telangiectasia patient and carrier erythroid cells to induced pluripotent stem cells. Stem Cell Res..

[120] Leeson H.C., Hunter Z., Chaggar H.K., Lavin M.F., Mackay-Sim A., Wolvetang E.J. (2021). Ataxia Telangiectasia iPSC line generated from a patient olfactory biopsy identifies novel disease-causing mutations. Stem Cell Res..

[121] Erceg S., Lukovic D., Moreno-Manzano V., Stojkovic M., Bhattacharya S.S. (2012). Derivation of Cerebellar Neurons from Human Pluripotent Stem Cells. Curr. Protoc. Stem Cell Biol..

[122] Muguruma K., Nishiyama A., Kawakami H., Hashimoto K., Sasai Y. (2015). Self-Organization of Polarized Cerebellar Tissue in 3D Culture of Human Pluripotent Stem Cells. Cell Rep..

[123] Nayler S., Agarwal D., Curion F., Bowden R., Becker E.B.E. (2021). High-resolution transcriptional landscape of xeno-free human induced pluripotent stem cell-derived cerebellar organoids. Sci. Rep..

[124] Lynch D.R., Chin M.P., Delatycki M.B., Subramony S.H., Corti M., Hoyle J.C., Boesch S., Nachbauer W., Mariotti C., Mathews K.D. (2020). Safety and Efficacy of Omaveloxolone in Friedreich Ataxia (MOXIe Study). Ann. Neurol..

[125] Lynch D.R., Perlman S., Schadt K. (2024). Omaveloxolone for the treatment of Friedreich ataxia: Clinical trial results and practical considerations. Expert Rev. Neurother..

[126] Subramony S.H., Lynch D.L. (2023). A Milestone in the Treatment of Ataxias: Approval of Omaveloxolone for Friedreich Ataxia. Cerebellum.

[127] Ristori G., Romano S., Visconti A., Cannoni S., Spadaro M., Frontali M., Pontieri F.E., Vanacore N., Salvetti M. (2010). Riluzole in cerebellar ataxia: A randomized, double-blind, placebo-controlled pilot trial. Neurology.

[128] Romano S., Coarelli G., Marcotulli C., Leonardi L., Piccolo F., Spadaro M., Frontali M., Ferraldeschi M., Vulpiani M.C., Ponzelli F. (2015). Riluzole in patients with hereditary cerebellar ataxia: A randomised, double-blind, placebo-controlled trial. Lancet Neurol..

[129] Ghanekar S.D., Kuo S.-H., Staffetti J.S., A Zesiewicz T. (2022). Current and emerging treatment modalities for spinocerebellar ataxias. Expert Rev. Neurother..

[130] Bye C.R., Qian E., Lim K., Daniszewski M., Garton F.C., Trần-Lê B.C., Liang H.H., Lin T., Lock J.G., Crombie D.E. (2025). Large-scale drug screening in iPSC-derived motor neurons from sporadic ALS patients identifies a potential combinatorial therapy. Nat. Neurosci..

